# Dual localization of receptor-type adenylate cyclases and cAMP response protein 3 unveils the presence of two putative signaling microdomains in *Trypanosoma cruzi*

**DOI:** 10.1128/mbio.01064-23

**Published:** 2023-07-21

**Authors:** Miguel A. Chiurillo, Joshua Carlson, Mayara S. Bertolini, Aqsa Raja, Noelia Lander

**Affiliations:** 1 Department of Biological Sciences, University of Cincinnati, Cincinnati, Ohio, USA; 2 Center for Tropical and Emerging Global Diseases, University of Georgia, Athens, Georgia, USA; 3 Department of Cellular Biology, University of Georgia, Athens, Georgia, USA; Albert Einstein College of Medicine, Bronx, New York, USA

**Keywords:** adenylate cyclase, CARP3, cell adhesion, contractile vacuole complex, cyclic AMP, flagellar distal domain, metacyclogenesis, osmoregulation, trypanosomes

## Abstract

*Trypanosoma cruzi* is the etiologic agent of Chagas disease, a leading cause of disability and premature death in the Americas. This parasite spends its life between a triatomine insect and a mammalian host, transitioning between developmental stages in response to microenvironmental changes. Among the second messengers driving differentiation in *T. cruzi*, cAMP has been shown to mediate metacyclogenesis and response to osmotic stress, but this signaling pathway remains largely unexplored in this parasite. Adenylate cyclases (ACs) catalyze the conversion of ATP to cAMP. They comprise a multigene family encoding putative receptor-type ACs in *T. cruzi*. Using protein sequence alignment, we classified them into five groups and chose a representative member from each group to study their localization (TcAC1–TcAC5). We expressed an HA-tagged version of each protein in *T. cruzi* and performed immunofluorescence analysis. A peculiar dual localization of TcAC1 and TcAC2 was observed in the flagellar distal domain and in the contractile vacuole complex (CVC), and their enzymatic activity was confirmed by gene complementation in yeast. Furthermore, TcAC1 overexpressing parasites showed an increased metacyclogenesis, a defect in host cell invasion, and a reduced intracellular replication, highlighting the importance of this protein throughout *T. cruzi* life cycle. These mutants were more tolerant to hypoosmotic stress and showed a higher adhesion capacity during *in vitro* metacyclogenesis, whereas the wild-type phenotype was restored after disrupting TcAC1 localization. Finally, TcAC1 was found to interact with cAMP response protein 3 (TcCARP3), co-localizing with this protein in the flagellar tip and CVC.

**IMPORTANCE:**

We identified three components of the cAMP signaling pathway (TcAC1, TcAC2, and TcCARP3) with dual localization in *Trypanosoma cruzi*: the flagellar distal domain and the CVC, structures involved in cell adhesion and osmoregulation, respectively. We found evidence on the role of TcAC1 in both cellular processes, as well as in metacyclogenesis. Our data suggest that TcACs act as signal sensors and transducers through cAMP synthesis in membrane microdomains. We propose a model in which TcACs sense the harsh conditions in the triatomine hindgut (nutrient deprivation, acidic pH, osmotic stress, ionic composition, hydrophobic interactions) and become active. Synthesis of cAMP then triggers cell adhesion prior completion of metacyclogenesis*,* while mediating a response to osmotic stress in the parasite. These results shed light into the mechanisms driving cAMP-mediated cell differentiation in *T. cruzi*, while raising new questions on the activation of TcACs and the role of downstream components of this pathway.

## INTRODUCTION

*Trypanosoma cruzi* is a protozoan parasite that causes Chagas disease, a zoonotic infectious disease considered a leading cause of disability and premature death in the Americas, where an estimate of six to seven million people are currently affected. The epidemiological pattern of Chagas disease changed in the last decades, and an increased number of cases has been reported in non-endemic countries of North America (USA and Canada), Europe, Africa, Middle East, and the Pacific (1). If untreated, this slow-progressing infection persists for a lifetime, causing severe cardiac disease in one third of the cases. However, most of the affected individuals remain undiagnosed and untreated. Understanding *T. cruzi* biology is crucial to find alternative approaches to control this silent disease. This Stercorarian trypanosome develops in the posterior gut of triatomine bugs and is transmitted to the mammalian host through the insect feces via skin wound or body mucosa. *T. cruzi* has a complex life cycle involving four major developmental stages that colonize very specific niches within its hosts, transitioning from one stage to another stage in response to environmental changes (reviewed in references 2 and 3). The epimastigote replicates in the triatomine midgut, and upon migration to the insect’s hindgut, it adheres to the rectal epithelium and differentiates into metacyclic trypomastigotes (4, 5). These forms infect the mammalian host, and after invading a host cell, they differentiate to amastigotes. After several rounds of replication, amastigotes transform into cell-derived trypomastigotes, which are released to the bloodstream and either invade other cells or are taken up by a triatomine. The signal transduction pathways driving differentiation in *T. cruzi* life cycle are still poorly understood (2, 6). 3′,5′-cyclic adenosine monophosphate (cAMP) is a small universal second messenger that relays the information from external stimuli into the intracellular environment, triggering cellular responses such as expression of a specific subset of genes, enzymatic activation, and differentiation. In mammalian cells, the basic molecular components of this signaling pathway are well established, and the expression of these proteins in different subcellular compartments determines spatiotemporal dynamics of cAMP signals (7, 8). Adenylate cyclases (ACs) catalyze the conversion of ATP to cAMP, while phosphodiesterases (PDEs) degrade cAMP, removing the intracellular signal. Canonical cAMP effectors EPAC (exchange protein directly activated by cyclic AMP), cyclic nucleotide-gated (cNMP-gated) ion channels, and protein kinase A (PKA) are either absent or cAMP unresponsive in *T. brucei* (9). In this parasite, cAMP has been found to mediate social motility (SoMo) and the mechanism to evade the mammalian host innate immune response (10 - 15). In *T. cruzi*, cAMP plays a role in metacyclogenesis (16 - 22) and osmoregulation (23 - 26). However, these signal transduction pathways remain largely unexplored in trypanosomes. Trypanosome ACs are transmembrane proteins that dimerize to become catalytically active (2, 6, 22, 27 - 29). In *T. cruzi*, these enzymes comprise a multigenic family of putative receptor-type adenylate cyclases (22, 29), but their individual localization and function remains unknown. One of them (TczAC) has been found to interact with the paraflagellar rod protein and to become active upon dimerization (30). In addition, antibodies raised against the catalytic domain of ACs label the flagellum and the flagellar pocket of *T. cruzi* metacyclic trypomastigotes (22). Two possible cAMP effectors have been identified in *T. cruzi*, PKA (31 - 33) and cAMP response proteins (CARPs) (34). Orthologs of other putative cAMP effectors have not been identified in the genome of *T. cruzi*. An *in silico* analysis identified several cyclic nucleotide monophosphate-binding proteins in *T. cruzi*, and at least one of them was shown to bind cAMP *in vitro* (TcCARP1) (34). CARPs are trypanosome-specific proteins that could play a role in a PKA-independent cAMP signaling pathway (11, 12, 27, 35, 36). Two recent studies have recognized CARP3 as a multi-adenylate cyclase regulator that plays a role in social motility and chemotaxis in *T. brucei* (11, 36). In this study, we identified three components of the cAMP signaling pathway (TcAC1, TcAC2, and TcCARP3) in two different subcellular compartments of *T. cruzi*. Our functional characterization of TcAC1 suggests that this protein interacts with TcCARP3 and synthesizes cAMP in two putative signaling domains: the contractile vacuole complex and the distal flagellar domain (flagellar tip). Our results provide evidence on the role of cAMP in the parasite’s ability to sense environmental cues such as nutrient deprivation, osmotic stress, and cell contact to the vector’s hindgut epithelium, mediating cell adhesion, metacyclogenesis, and response to osmotic stress in *T. cruzi*.

## RESULTS

### Adenylate cyclases comprise a conserved gene family in *Trypanosoma cruzi*

**Fig 1:**
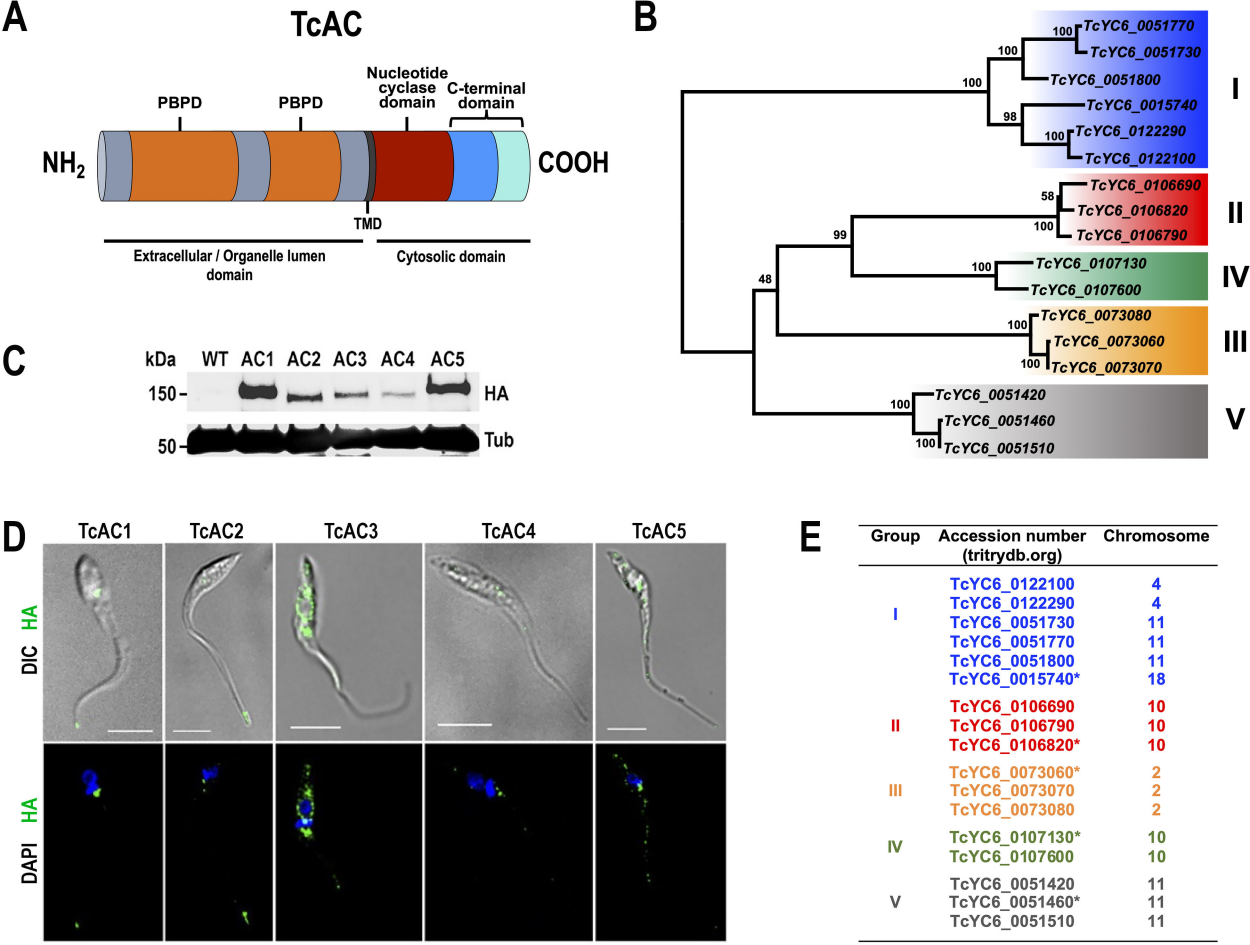
Analysis of TcAC family. (**A**) General topology of *T. cruzi* adenylate cyclases. The C-terminal domain can be divided in a proximal region (blue) and a less-conserved distal region among TcAC groups (light blue). One or two periplasmic binding protein domains (PBPD, orange) are identified upstream a single transmembrane domain (TMD, black). Downstream the TMD, a conserved nucleotide cyclase domain is shown in burgundy color. The predicted extracellular and cytosolic domains are indicted bellow each region of TcAC topology. In an endomembrane localization, the extracellular domain is expected to be facing the lumen of the organelle. (**B**) Phylogenetic tree of TcAC amino acid sequences. Phylogenetic tree was constructed using the Neighbor-Joining method with 17 full-length predicted TcAC proteins from *T. cruzi* YC6 genome found in tritrypdb.org, as described in Materials and Methods. The tree is drawn to scale, with branch lengths in the same units as those of the evolutionary distances used to infer the phylogenetic tree. Scale is in units of the number of amino acid substitutions per site. Sequences are indicated with TriTrypDB gene IDs. Roman numbers on the right and shading colors indicate the group of TcAC. (**C**) Western blot analysis of total protein extracts from *T. cruzi* epimastigotes wild type (WT), and overexpressing HA-tagged TcAC1–TcAC5 (expected sizes: 144.0, 142.5, 139.4, 139.8, and 141.5 kDa for AC1, AC2, AC3, AC4, and AC5, respectively), using anti-HA antibodies. Anti-α-tubulin antibodies were used as a loading control. (**D**) Immunofluorescence analysis of HA-tagged TcAC1–TcAC5 overexpressing epimastigotes using anti-HA antibodies (green). A differential interference contrast (DIC) image is shown in the upper panel. The lower panel shows merge images of HA labeling (TcACs) and DAPI labeling (nucleus and kinetoplast, blue). Scale bars: 5 μm. (**E**) TcAC genes encoding putative receptor-type adenylate cyclases found in *T. cruzi* YC6 genome. Different colors were assigned to each group: group I (blue), group II (red), group III (yellow), group IV (green), and group V (gray). The chromosome location for each gene is shown at the right column. Asterisks within each group indicate the genes that were chosen for cloning and overexpression in *T. cruzi*.

Trypanosomal ACs are structurally unique proteins, containing a single transmembrane domain, a conserved catalytic domain located at the C-terminus of the protein, and a large variable extracellular domain, resembling mammalian receptor-type guanylyl cyclases (27). These proteins are encoded by genes that comprise multigene families (29). Figure 1A shows the general topology of TcACs. In *T. cruzi*, there are 17 open reading frames annotated as putative receptor-type adenylate cyclases in the Y strain genome (Fig. 1E). In addition, we identified other 26 sequences, that after manually curation corresponded to 15 AC pseudogenes and truncated copies (Table S1). The 17 TcAC genes share an overall amino acid identity of 57.6%–99.9% (Fig. S1). Using protein sequence alignment, we classified them into five different groups (TcAC1–TcAC5), within which TcACs are highly conserved (85.1%–99.9% of amino acid identity). The amino acid sequence identity among TcAC groups I to V was found to be lower within the N-terminal and C-terminal regions (52.2%–75.1% and 25.4%–73.2%, respectively) (Fig. S1). As expected, the predicted AC catalytic domain displays the highest amino acid identity among the five groups (84.8%–95.2%). A neighbor-joining phylogenetic tree including the 17 TcACs supports their classification in five groups (Fig. 1B). This phylogenetic tree shows two main branches, one of them clustering TcAC group I and the other one clustering groups II to V. These results are consistent with the two major TcAC subclasses described by Hamedi et al. (22). In this analysis, when we included the three TcAC sequences from *T. cruzi* reference strain CL Brener that have been previously characterized, ADC1 (GenBank AJ012096), ADC4 (GenBank AJ011684), and TczAC (GenBank AF040382) (30, 37), each one of them perfectly fit into one of the five groups (Fig. S2).

The genomic distribution of the 17 AC genes in the *T. cruzi* YC6 *in silico* assembled chromosomes (data from tritrypdb.org) is detailed in Fig. S3. Interestingly, TcAC group I genes are distributed in three different chromosomes (chromosomes 4, 11, and 18), whereas TcAC group II and IV genes are found in two different loci within the same chromosome (chromosome 10), which is consistent with the fact that TcAC group II and IV stem from the same branch in the phylogenetic tree (Fig. 1B). Commonly, TcAC genes are found in clusters with other repetitive sequences that could be involved in gene duplication events and AC copy dispersal in the *T. cruzi* genome.

To analyze the AC family organization in other *T. cruzi* strains, we performed phylogenetic analyses of AC amino acid sequences from *T. cruzi* YC6 (DTU-TcII), Dm28c (DTU-TcI), and TCC (DTU-TcIV) strains whose genome sequences were obtained by PacBio and Illumina sequencing technologies. This phylogenetic tree confirms that the AC multigene family in *T. cruzi* that can be clustered into five groups (Fig. S4). Interestingly, whereas in the *T. cruzi* CL Brener strain genome (obtained by Sanger sequencing) (38), there are only six full-length AC genes (considering both Esmeraldo and non Esmeraldo-like haplotypes); in *T. cruzi* YC6, Dm28c, and TCC strains, we found 17, 11, and 20 full-length genes, respectively. The apparent low number of TcAC genes in the CL Brener hybrid genome (DTU-TcVI) could be explained by the repetitive nature of the *T. cruzi* genome, and by the DNA sequencing and genome assembly technology used in 2005, which produced the extensive collapse of repetitive regions, resulting in an underestimation of the copy number and an imprecise assignment of the organization of several multigene families (39, 40). In this study, further characterization of TcACs was performed using *T. cruzi* Y strain parasites.

### TcAC1 shows dual localization in the replicative stages of *T. cruzi* life cycle

**Fig 2:**
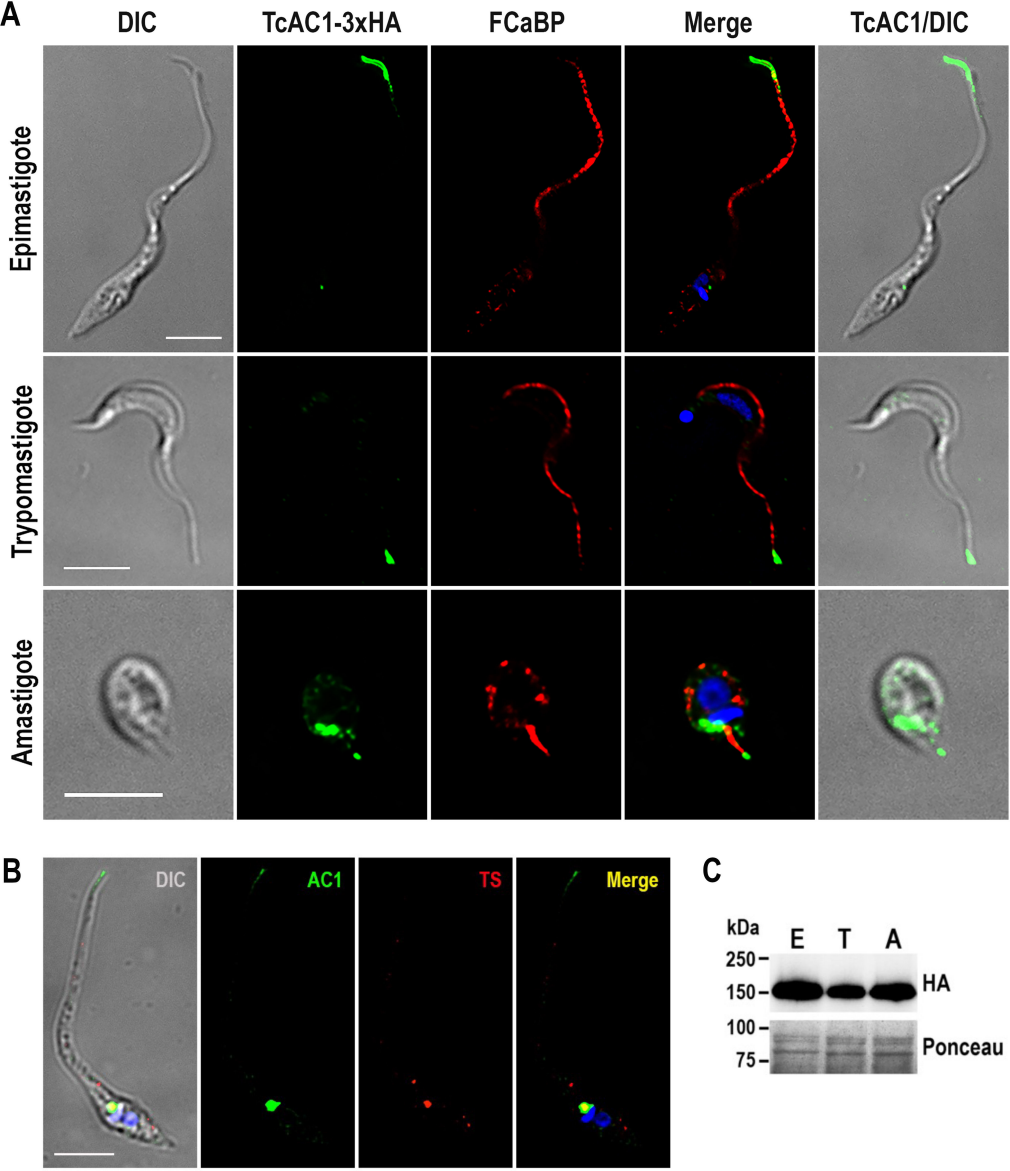
TcAC1 localization. (**A**) Immunofluorescence microscopy of *T. cruzi* parasites overexpressing TcAC1-3xHA in different developmental stages: epimastigote, trypomastigote, and amastigote. From left to right: the images show differential interference contrast (DIC), TcAC1-3xHA (green), flagellar calcium binding protein (FCaBP, red), merged image including DAPI labeling of nucleus and kinetoplast (blue), and merged DIC and HA labeling. (**B**) Immunofluorescence microscopy of *T. cruzi* epimastigote in stationary phase of growth overexpressing TcAC1-3xHA under hypoosmotic conditions. From left to right: differential interference contrast (DIC), TcAC1-3xHA (green), *T. cruzi* trans-sialidase (TcTS, red), merged image including DAPI labeling of nucleus and kinetoplast (blue). Co-localization of TcAC1 and TcTS can be observed (yellow). Scale bars: 5 μm. (**C**) Western blot analysis of TcAC1-3xHA overexpressing parasites in different developmental stages: epimastigote (**E**), trypomastigote (**T**), and amastigote (**A**). Ponceau staining of the blot is included as loading control.

**Fig 3:**
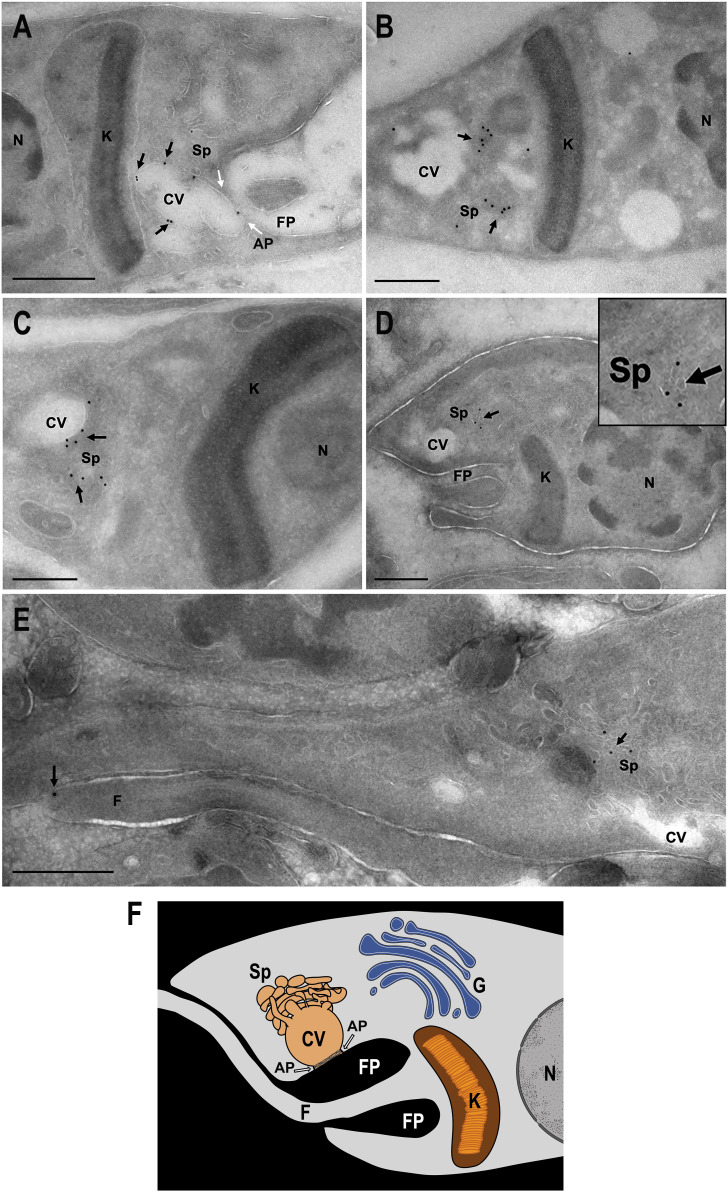
Cryo-immunoelectron microscopy of TcAC1-3xHA overexpressing parasites. TcAC1-3xHA was detected in the bladder of the contractile vacuole (CV) and in the spongiome (Sp) of the CVC in *T. cruzi* epimastigotes (**A–C**) and amastigotes (**D**) using anti-HA antibodies and gold-conjugated anti-rabbit secondary antibody (18 nm). The dual localization of TcAC1 (flagellar tip and spongiome of the CVC) was also detected in an epimastigote (**E**). The inset in **D** shows the detail of TcAC1 labeled at the spongiome. A schematic representation (relative spatial localization) of the structures observed in these micrographs is shown in **F**. Gold particles labeling TcAC1 are indicated by arrows. Other structures: nucleus (N), kinetoplast (K), flagellar pocket (FP), Golgi (G). Scale bars: 500 nm.

To study the individual role of *T. cruzi* adenylate cyclases, we chose a representative member from each group to express a tagged version of them: TcAC1, TcAC2, TcAC3, TcAC4, and TcAC5 (TriTrypDB gene IDs: TcYC6_0015740, TcYC6_0106820, TcYC6_0073060, TcYC6_0107130, and TcYC6_0051460). TcAC1 is the group I member exhibiting the highest similarity to previously characterized ADC4 (37), while TcAC2 is the group II member presenting the highest similarity to TczAC (30) (Fig. S2). TcAC3–TcAC5 were randomly chosen within TcAC groups III, IV, and V. Gene knockout or endogenous tagging of specific TcACs by standard CRISPR/Cas9 methods used in our lab (41 - 43) was not possible due to the high sequence identity observed within members of each TcAC group, including the UTR regions flanking these genes. This made impossible the selection of specific protospacers and homology regions to target and replace or tag each one of these genes. Alternatively, to establish their cellular localization, we overexpressed an HA-tagged version of each open reading frame in *T. cruzi* epimastigotes. Overexpression in clonal populations was confirmed by western blot analysis (expected sizes: 144.0, 142.5, 139.4, 139.8, and 141.5 kDa for TcAC1–TcAC5, respectively) (Fig. 1C). Their cellular localization was determined by immunofluorescence analysis (IFA) using anti-HA antibodies. IFA results show a peculiar dual localization pattern of TcAC1 and TcAC2 in the flagellar distal domain and in the contractile vacuole complex (CVC), while TcAC3 seems to partially localize to the ER, TcAC4 to the CVC, and TcAC5 accumulates in the CVC but is also spread over the cell body in a punctate pattern (Fig. 1D). The dual localization pattern of TcAC1 was clearly observed in the replicative stages of *T. cruzi* (epimastigotes and amastigotes), while in cell-derived trypomastigotes, TcAC1 localized mainly to the flagellar tip, with some faint CVC labeling (Fig. 2A). Antibodies anti-flagellar calcium binding protein (FCaBP) were used as a flagellar marker in these IFAs. To confirm the CVC localization of TcAC1, we performed IFA of epimastigotes in stationary phase of growth under hypoosmotic conditions, inducing the swelling of the contractile vacuole bladder to facilitate the visualization of this organelle. Co-localization of TcAC1 with a trans-sialidase (TcTS, a GPI-anchored protein that localizes to the CVC in differentiating epimastigotes) was confirmed by IFA (Fig. 2B). The expression of TcAC1 was confirmed by western blot analysis in different *T. cruzi* developmental stages (Fig. 2C). IFA of TcAC1 overexpressing trypomastigotes under hypoosmotic conditions confirmed the presence of the protein in the flagellar tip and CVC of this infective stage (Fig. S5A). Because this dual localization pattern (flagellar tip and CVC) was never observed before in a *T. cruzi* protein, TcAC1 localization was verified at the ultrastructural level by cryo-immunoelectron microscopy. TcAC1-3xHA was detected with an anti-HA polyclonal antibody and a gold-conjugated anti-rabbit IgG secondary antibody. TcAC1 localization to the CVC bladder (Fig. 3A) and spongiome (Fig. 3B through E) was confirmed in *T. cruzi* epimastigotes (Fig. 3A through C) and amastigotes (Fig. 3D). Figure 3E shows the dual localization of TcAC1 at the flagellar tip and the CVC spongiome, as indicated by arrows. TcAC1 was not detected at the flagellar pocket in any of the analyzed images. A schematic representation of the structural organization observed in these micrographs is shown in Fig. 3F.

To verify the orientation of the protein in the plasma membrane, we performed IFA of TcAC1-3xHA overexpressing epimastigotes under hypoosmotic stress with and without permeabilizing the cells. No labeling was detected in non-permeabilized parasites (Fig. S5B), confirming the cytosolic orientation of the C-terminal region of the protein, containing the catalytic domain. Concomitantly, we expect a cytosolic orientation for the catalytic domain of TcAC1 in the CVC, but further verification is required.

Finally, localization of TcAC3 to the ER was verified by expressing an alternative C-terminal tagged version of the protein (TcAC3-3xc-Myc) in *T. cruzi* epimastigotes, and antibodies anti-TbBiP (an ER-resident protein) (44) have been successfully used in *T. cruzi* (42). These results showed partial co-localization of TcAC3 with the ER marker (Fig. S5C), confirming the peculiar localization of this protein, with respect to the other four TcACs analyzed. However, we cannot rule out that this ER localization results from placing the tag in the C-terminus of TcAC3 while overexpressing it, which should be further investigated by expressing an N-terminal tagged version of the protein. Expression of TcAC3-3xc-Myc was confirmed by western blot analysis (Fig. S5D).

### TcAC1 and TcAC2 are catalytically active adenylate cyclases

**Fig 4:**
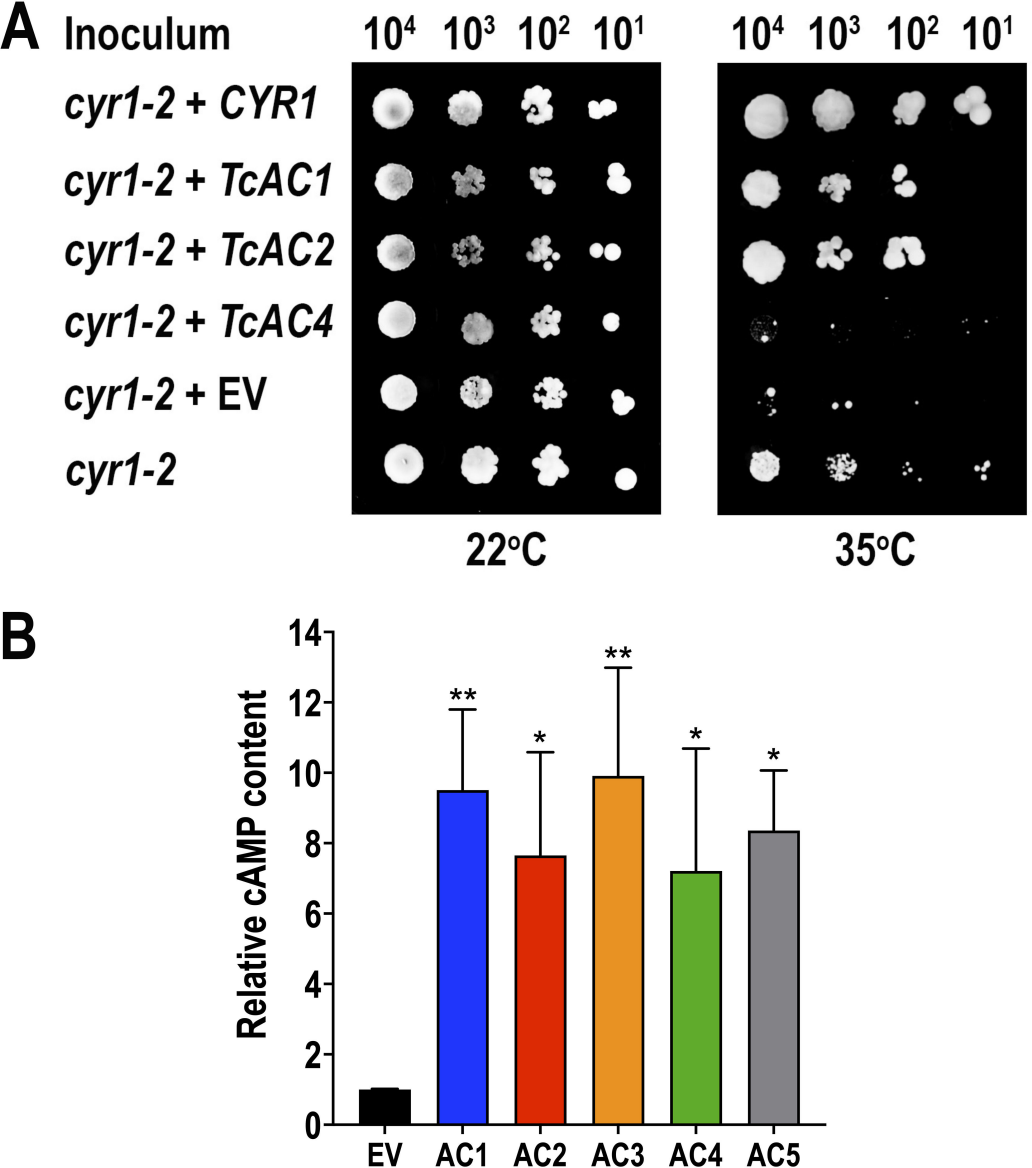
Enzymatic activity of TcACs. (**A**) Gene complementation in yeast. A temperature-sensitive *S. cerevisiae* AC mutant (*cyr1-2*) was transformed with a yeast expression vector containing either the yeast wild-type AC (*CYR1*) or the genes encoding TcAC1, TcAC2, and TcAC4. An empty vector (EV) was transformed as a control, and yeast viability was assessed at the permissive (22°C) and restrictive (35°C) temperatures. TcAC3 and TcAC5 could not be expressed in yeast. (**B**) cAMP content was measured in *T. cruzi* epimastigotes overexpressing TcAC1–TcAC5, relative to the content of the empty vector control parasites (EV). The results are expressed as mean ± SD (*n* = 3). *P < 0.05; **P < 0.01 [one-way analysis of variance (ANOVA) with Dunnett’s multiple comparisons test].

To determine if these five TcACs (TcAC1–TcAC5) are catalytically active, we performed gene complementation experiments in yeast. A temperature-sensitive *Saccharomyces cerevisiae* AC mutant (*cyr1-2*) was transformed with an expression vector containing either the yeast wild-type AC gene (*CYR1*) or each one of the genes encoding *TcAC1–TcAC5*. An empty vector (EV) was transformed as a control, and yeast viability was assessed at the permissive (22°C) and restrictive (35°C) temperatures. The activity of TcAC1 and TcAC2 was confirmed by gene complementation, while TcAC4 did not restore the wild-type AC phenotype (Fig. 4A). TcAC3 and TcAC5 could not be expressed in yeast, as shown by western blot analysis (Fig. S5E). However, the cAMP content in all five AC overexpression mutants (TcAC1–TcAC5) was significantly higher than that in the EV control in total protein extracts of epimastigotes (Fig. 4B), suggesting that they are all catalytically active in *T. cruzi*. Because AC enzymatic activity was confirmed for TcAC1 and TcAC2 by gene complementation, and they display the same localization pattern, for additional characterization, we focused on the phenotypic analysis of TcAC1 overexpressing parasites.

### TcAC1 plays a role in different developmental stages

**Fig 5:**
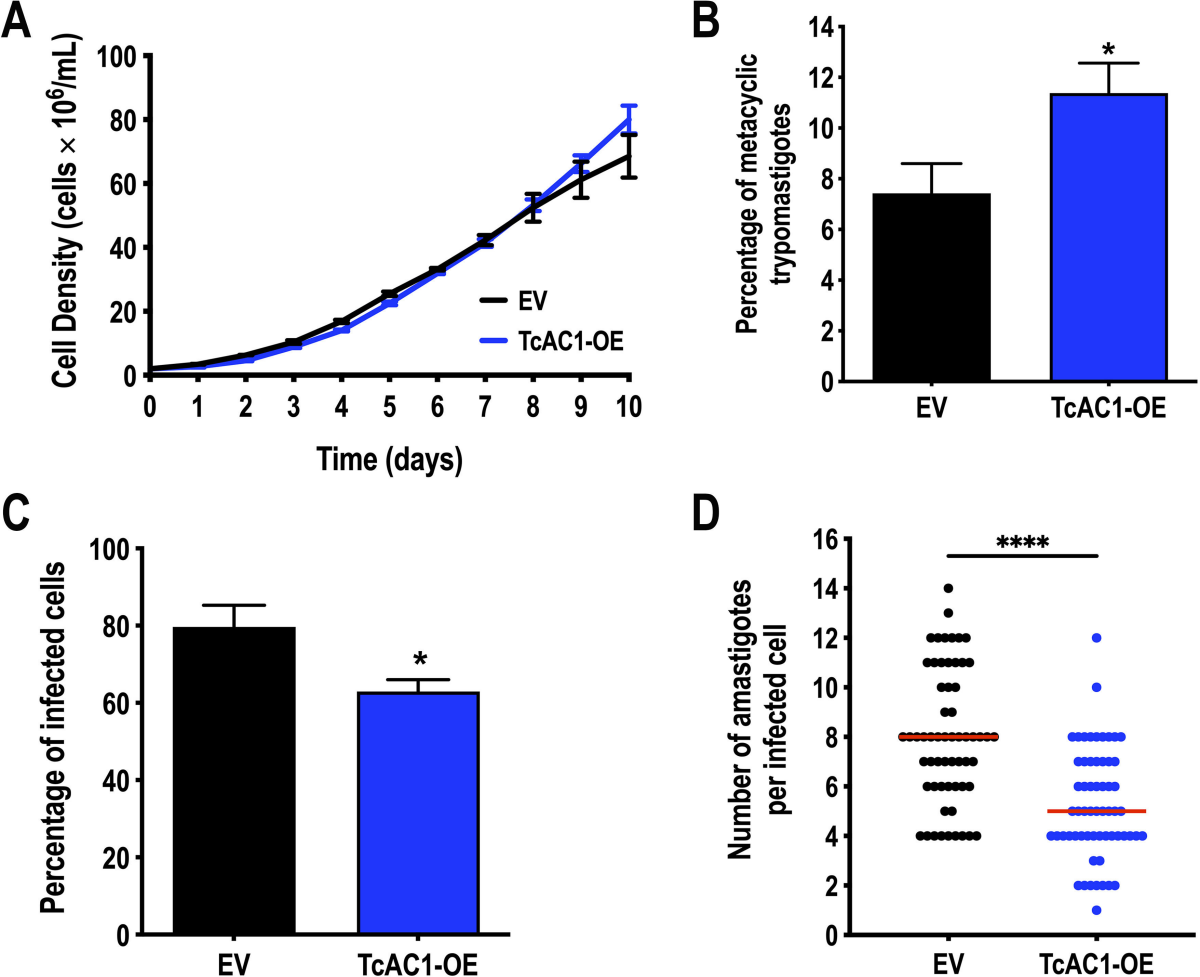
Phenotype of TcAC1 overexpressing parasites. (**A**) Growth of TcAC1-OE and empty vector (EV) control epimastigotes in LIT medium. (**B**) Percentage of metacyclic trypomastigotes observed after incubation of epimastigotes in TAU 3AAG medium. Differentiation of epimastigotes to metacyclic trypomastigotes was quantified by DAPI staining to distinguish the position of the kinetoplast related to the nucleus by fluorescence microscopy. (**C**) Invasion assay using *T. cruzi* trypomastigotes to infect Vero cells. The percentage of infected cells was assessed 4 hours post infection by fluorescence microscopy. Values are mean ± SD; *n* = 3; *P < 0.05 (Student’s *t*-test). (**D**) Intracellular replication of amastigotes 48 hours post infection. Red lines correspond to the medians from one representative experiment (*n* = 60 per cell line). ****P < 0.0001 (Mann-Whitney test). Three experiments were performed, and the results were reproducible.

An increase in the intracellular levels of cAMP has been previously reported during *in vitro* metacyclogenesis in *T. cruzi* (16 - 22 - 22). To further investigate the role of cAMP in *T. cruzi* life cycle, we analyzed the phenotype of TcAC1 overexpressing parasites (TcAC1-OE) in different developmental stages. Our results indicate that TcAC1-OE epimastigotes exhibit no growth phenotype but increased metacyclogenesis *in vitro* (Fig. 5A and B), while cell-derived trypomastigotes showed a defect in the invasion of mammalian cells (Fig. 5C) and a reduced intracellular replication of amastigotes was observed in Vero cells (Fig. 5D), highlighting the importance of TcAC1 throughout *T. cruzi* life cycle.

### The localization pattern of TcAC1 is required for cell adhesion and metacyclogenesis

**Fig 6:**
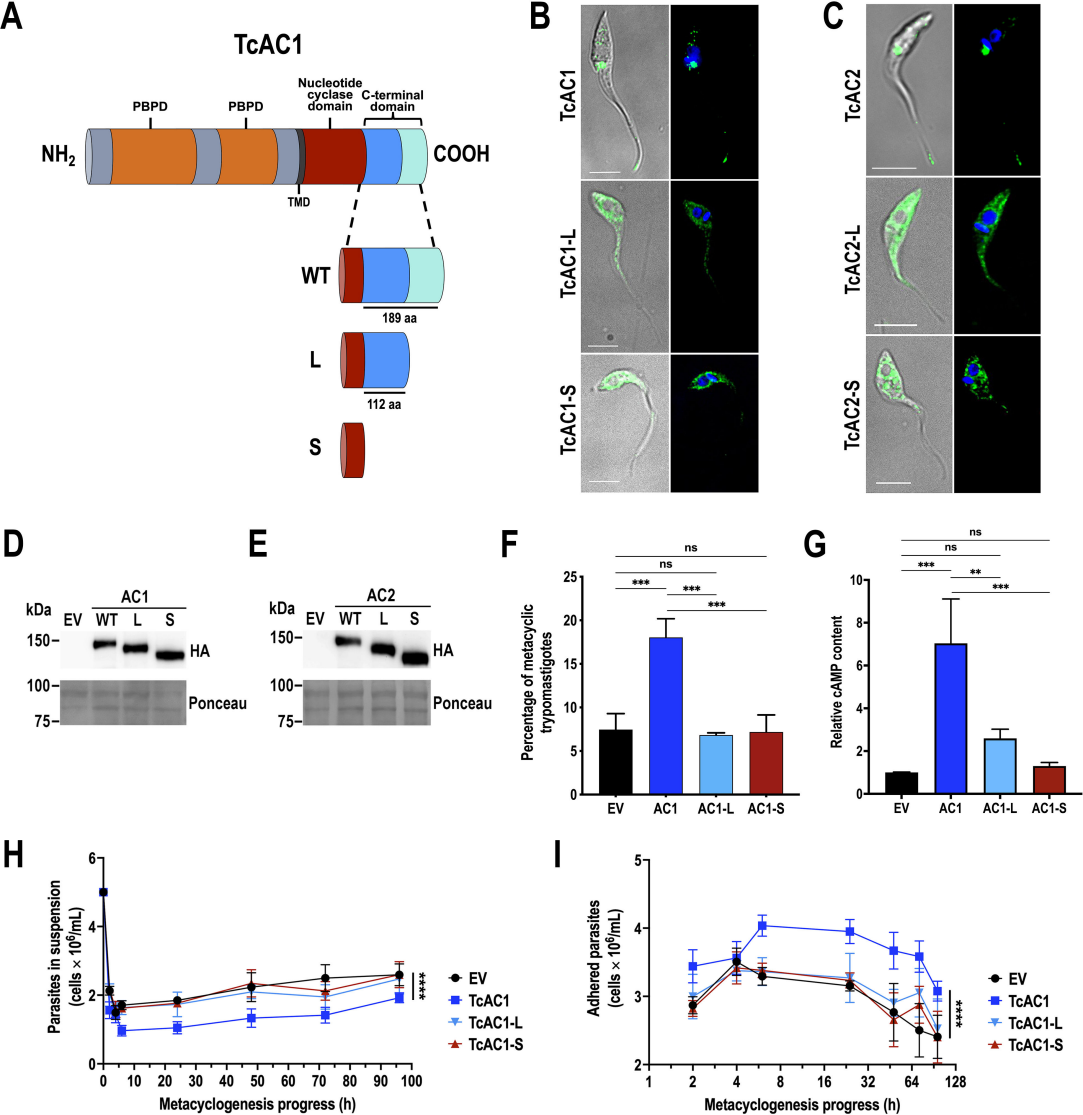
Disruption of dual localization pattern of TcAC1 and TcAC2. (**A**) Schematic representation of TcAC1 topology and the generation of TcAC1-L and S mutants. The C-terminal domain can be divided in a proximal region (112 aa, blue) and a less-conserved (among TcAC groups) distal region (77 aa, light blue). Two periplasmic binding protein domains (PBPD, orange) are identified upstream a single transmembrane domain (TMD, black). Downstream the TMD, a conserved nucleotide cyclase domain is shown in burgundy color. In the short mutant (S), the last 171 residues of TcAC1 were removed, while in the long mutant (L), only the less-conserved C-terminal distal region (77 aa) was removed. (**B**) IFA of *T. cruzi* epimastigotes overexpressing TcAC1 and its truncated versions TcAC1-L and TcAC1-S. The three proteins were tagged with 3xHA and were detected by fluorescence microscopy using anti-HA antibodies (green). (**C**) Same experiment as in B, but with TcAC2 overexpressing epimastigotes and its mutant versions TcAC2-L and TcAC2-S (green labeling). Nucleus and kinetoplast were stained with DAPI (blue). Differential interference contrast images are shown on the left panels of **B** and **C**. (**D**) and (**E**) Western blot analyses of empty vector (EV), wild type (WT, and parasites expressing TcAC1, TcAC1-L, and TcAC1-S, or TcAC2, TcAC2-L, and TcAC2-S using total protein extracts and anti-HA antibodies. Ponceau red staining is shown as loading control. (**F**) Percentage of metacyclic trypomastigotes observed after incubation of epimastigotes in TAU 3AAG medium. Percentage of metacyclic trypomastigotes was quantified by DAPI staining to distinguish the position of the kinetoplast related to the nucleus by fluorescence microscopy. (**G**) cAMP content of TcAC1, TcAC1-L, and TcAC1-S cell lines relative to control (EV) parasites. In **F** and **G**, values are mean SD; *n* = 3; **P < 0.01; ***P < 0.001; ns, not significant differences with respect to EV cells (one-way ANOVA with Dunnett’s multiple comparisons test). (**H**) Time course of parasite density during *in vitro* metacyclogenesis in TAU 3AAG medium. (**I**) Time course of adhered parasites throughout *in vitro* metacyclogenesis in TAU 3AAG medium. In **H** and **I**, values are mean SD; *n* = 3; ****P < 0.0001 (two-way ANOVA with Dunnett’s multiple comparisons test, comparing mean values of each cell line with the EV control, main column effect).

The analysis of amino acid identity among the 17 AC genes present in *T. cruzi* YC6 genome (Fig. S1 and 6) indicates that the C-terminal domain displays the highest degree of divergence between the five TcAC groups. However, the amino acid identity among ACs from the same group expands from 80% to 100%, with more than 95% identity within each of the TcAC groups II to V. Therefore, we hypothesized that the C-terminal domain is important to target TcACs to their specific subcellular compartments. To study the biological relevance of the localization pattern observed in TcAC1 and TcAC2, truncated versions of these proteins (TcAC1-L, TcAC1-S, TcAC2-L, and TcAC2-S) were expressed in *T. cruzi* epimastigotes, as illustrated in Fig. 6A and Fig. S7A. To generate these mutants, we amplified truncated TcAC1 and TcAC2 genes in which either the distal and less-conserved C-terminal region between TcAC1 or TcAC2 was deleted (aa 1204–1281 and aa 1178–1278 for AC1 and AC2, respectively, L mutants), or the entire region downstream the catalytic domain of these proteins was removed (aa 1110–1281 and aa 1088–1278 for AC1 and AC2, respectively, S mutants). The truncated fragments were cloned into pTREX-n-3xHA expression vector and used to transfect *T. cruzi* wild-type epimastigotes. Then, the expression and localization of TcAC1-L (135.2 kDa), TcAC1-S (124.8 kDa), TcAC2-L (131.6 kDa), and TcAC2-S (121.8 kDa) were analyzed by IFA and western blot (Fig. 6B through E). Although no obvious sequence similarities between the distal C-terminal region of TcAC1 and TcAC2 proteins were observed, deletion of this segment was sufficient to disrupt the dual localization pattern of both enzymes (Fig. 6B and C). This result is consistent with the disruption of the flagellar localization observed in *T. brucei* ACP1 and ACP2 when the last 45 and 46 C-terminal amino acids were deleted, respectively (45). However, it has been proposed that endocytic motifs, such as YXXΦ, YXXXΦ, and [D/E]XXXL[L/I], are involved in targeting proteins to the CVC (46 - 48). Some of these signals were identified in the C-terminal domain of TcACs (Fig. S7A). Therefore, we would expect that only in the S mutants (where these signals are absent), the dual localization pattern of TcAC1 and TcAC2 was disrupted but not in the L mutants, where these signals are still present. However, our results indicate that deletion of the distal C-terminal region in L mutants is sufficient to disrupt the localization of TcAC1 and TcAC2, suggesting that unknown targeting elements are present in the distal C-terminal domain. Another possibility is that deletion of the distal C-terminal region in L mutants is inducing conformational changes or protein misfolding that affect the still present endocytic motifs in the proximal region as targeting signals. A third possibility is that ER exit signals present in the distal C-terminal region prevent them to complete this step, leading to TcAC1/2 retention in the ER. We performed IFA of TcAC1 (L and S) mutants using anti-HA and anti-BiP antibodies. Our results indicate that both truncated mutants exhibit a cytosolic pattern with partial ER localization (Fig. S7B), confirming the disruption of TcAC1 dual localization in L and S mutants.

**Fig 7:**
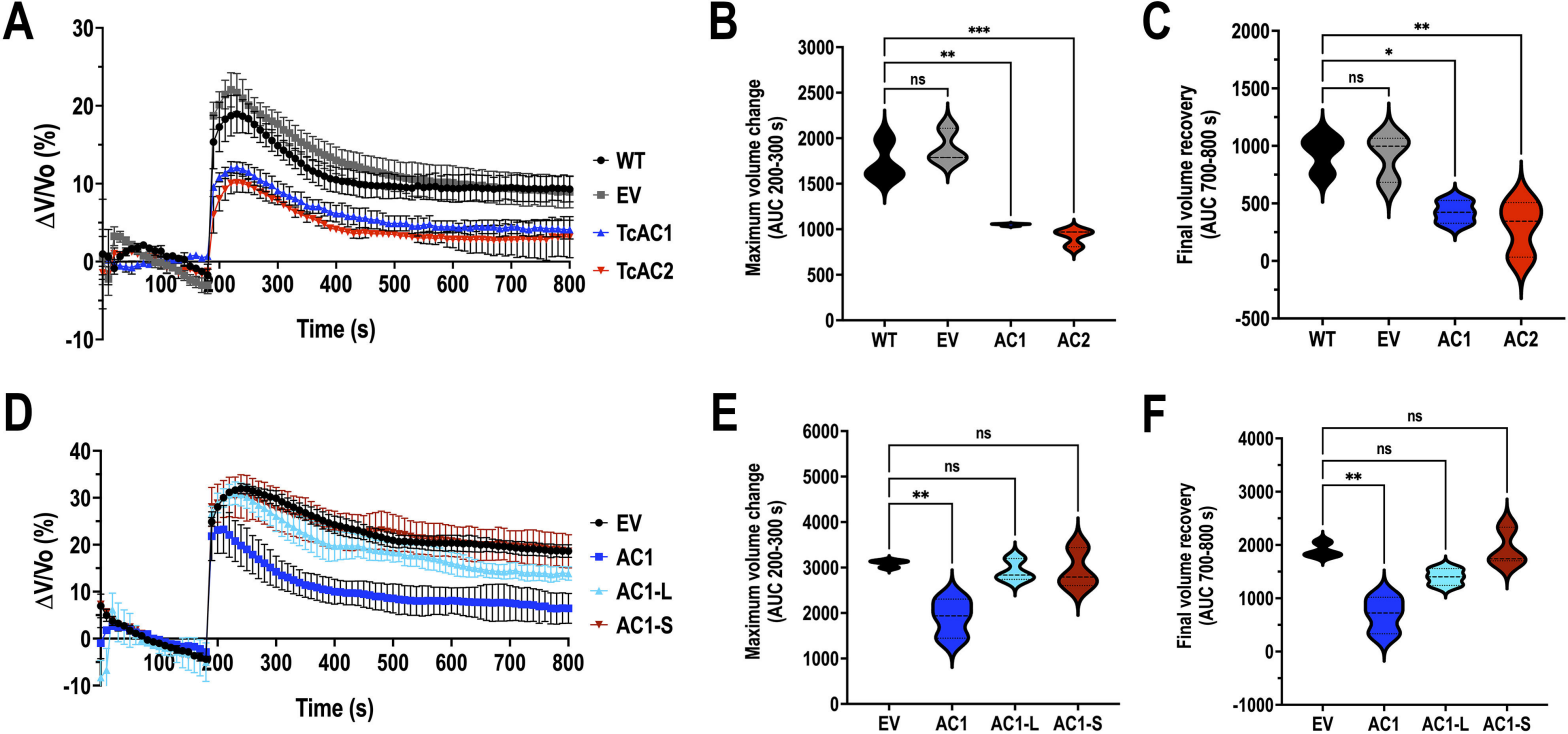
Regulatory volume decrease (RVD) upon hypoosmotic stress in TcAC mutants. (**A**) The light scattering pattern of epimastigotes suspended in isosmotic buffer was recorded for 120 seconds and diluted to a final osmolarity of 115 mOsm/L under constant ionic conditions. Relative changes in cell volume were monitored by determining absorbance at 550 nm over time in wild type (WT), empty vector (EV), TcAC1, and TcAC2 overexpressing parasites. The absorbance values were normalized by the initial volume under isosmotic conditions and expressed as percentage of volume change. (**B**) Analysis of the maximum volume change under hypoosmotic conditions. The area under the curve (AUC) in **A** was calculated between 200 and 300 seconds for all cell lines. (**C**) Final volume recovery calculated as the area under the curve in **A** between 700 and 800 seconds. Values are mean SD; *n* = 3; *P < 0.05; **P < 0.01; ***P < 0.001; ns, not significant differences with respect to WT cells (one-way ANOVA with Dunnett’s multiple comparisons test). (**D**), (**E**), and (**F**) Same experiments as in **A**, **B**, and **C**, but using cell lines empty vector (EV), TcAC1, TcAC1-L, and TcAC1-S overexpressing parasites. Values are mean SD; *n* = 3; **P < 0.01; ns, not significant differences with respect to EV cells (one-way ANOVA with Dunnett’s multiple comparisons test).

Because the localization pattern was similarly disrupted in both proteins, we performed further phenotypical analyses only with TcAC1. In this regard, the increased metacyclogenesis observed in TcAC1 overexpressing parasites was reversed by these deletions to the normal values of the empty vector (EV) control (Fig. 6F), highlighting the importance of TcAC1 localization to complete the differentiation process. The cAMP content in TcAC1-L and S mutants was not significantly different to that of the control cells, but significantly lower than that of TcAC1 overexpressing parasites (Fig. 6G) indicating that the truncated versions are enzymatically inactive, probably because they have lost their native conformation at the flagellar tip and CVC membranes. Moreover, during the *in vitro* metacyclogenesis process, a time course of the parasite density in TAU 3AAG medium was determined by counting the number of cells in suspension at different time points (Fig. 6H). This value is inversely proportional to the number of parasites adhered to the flask. Adhesion is a step necessary to complete the differentiation of epimastigotes into infective metacyclic trypomastigotes, *in vivo* and *in vitro* (4, 22). Our results indicate that TcAC1 overexpressing parasites possess an increased adhesion phenotype compared to control cells, while this effect was not observed in TcAC1-L and S mutants, suggesting a restitution of the normal adhesion phenotype (Fig. 6I), a process that occurs through the tip of the flagellum during *T. cruzi* metacyclogenesis (5).

### Disruption of TcAC1 and TcAC2 localization affects regulatory volume decrease under hypoosmotic stress

The role of cAMP in the parasite’s ability to recover cell volume under hypoosmotic conditions has been previously described in *T. cruzi* (23 - 26). To study the effect of TcAC1 and TcAC2 overexpression in the osmoregulatory capacity of epimastigotes, we evaluated regulatory volume decrease (RVD) after exposing the parasites to hypoosmotic stress, by following variations in absorbance over time, as described previously (49). The RVD capacity was quantified using two different parameters: the maximum cell volume change upon induction of hypoosmotic stress and the final volume recovery. TcAC1 and TcAC2 overexpressing parasites showed a significantly smaller change in cell volume than wild type and empty vector cells (Fig. 7A and B) and were more efficient in recovering their initial volume (Fig. 7A and C), evidencing in general a higher osmoregulatory capacity than control parasites. Considering the central role that the contractile vacuole complex plays in *T. cruzi* osmoregulation (2, 24, 47), we also evaluated RVD in TcAC1-S and TcAC1-L mutants compared with TcAC1 overexpressing and control parasites. Upon induction of hypoosmotic stress, TcAC1-L and TcAC1-S epimastigotes (in which TcAC1 no longer localizes to the contractile vacuole) showed a maximum cell volume change significantly higher than that of TcAC1 overexpressing parasites (Fig. 7D and E) and a less efficient recovery of their initial cell volume (Fig. 7D and F). In general, the enhanced RVD capacity observed in TcAC1-OE parasites was not detected in TcAC1-L and TcAC1-S mutants, which showed an osmotic stress response equivalent to that of control cells (Fig. 7D through F). Our results support the hypothesis that TcAC1 localization to the CVC is necessary to maintain the osmoregulatory capacity of the cell through cAMP synthesis in this putative microdomain.

### TcAC1 and TcCARP3 interact and co-localize in *T. cruzi* epimastigotes

**Fig 8:**
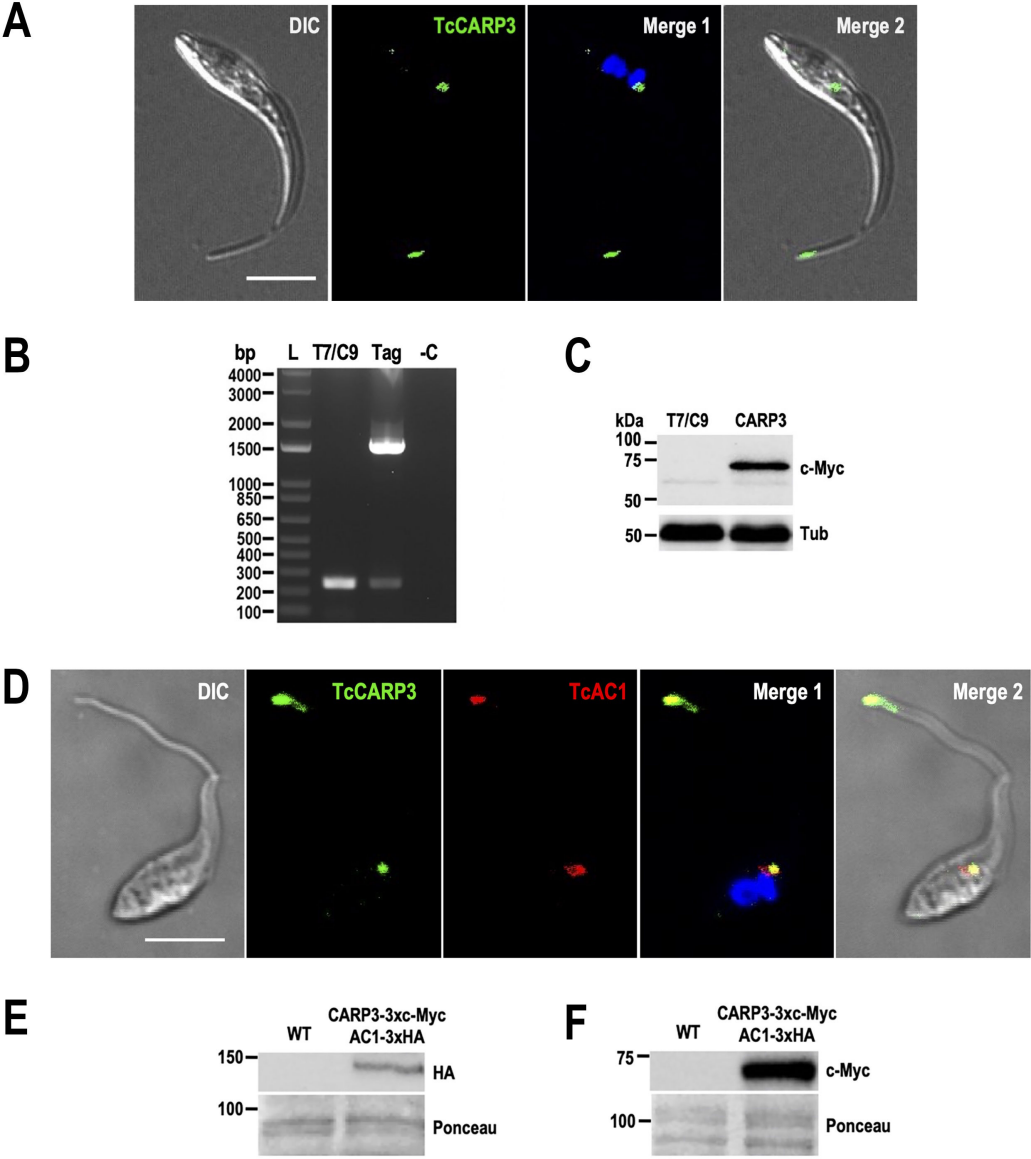
TcAC1 and TcCARP3 co-localization in two microdomains. (**A**) Immunofluorescence microscopy of TcCARP3-3xc-Myc endogenously tagged epimastigotes using anti-c-Myc antibodies. From left to right: differential interference contrast (DIC), TcCARP3-3xc-Myc (green), merged image with DAPI labeling of nucleus and kinetoplast (blue), and merged image with DIC. (**B**) PCR to verify CRISPR/Cas9-mediated TcCARP3 endogenous tagging. Lanes on 1% agarose gel: L, 1 kb plus ladder; T7/C9, parental cell line expressing T7 RNA polymerase and Cas9, Tag, c-Myc-tagged parasites; -C, PCR negative control. The expected size of PCR products was 244 and 1530 bp in T7/Cas9 and TcCARP3-3xc-Myc-tagged parasites, respectively. (**C**) Western blot analysis of T7/Cas9 and TcCARP3-3xc-Myc protein extracts, using anti anti-c-Myc antibodies. α-Tubulin (Tub) was used as loading control. (**D**) Immunofluorescence microscopy of TcCARP3-3xc-Myc/TcAC1-3xHA epimastigotes under hypoosmotic conditions, using anti-c-Myc and anti-HA antibodies. From left to right: differential interference contrast (DIC), TcCARP3-3xc-Myc (green), TcAC1-3xHA (red), merged image including DAPI labeling of nucleus and kinetoplast (blue), and merged image with DIC. Co-localization of TcAC1 and TcCARP3 can be observed at the CVC at the flagellar tip (yellow). Scale bars: 5 μm. (**E**) and (**F**) Western blot analyses of wild type (WT) and TcCARP3-3xc-Myc/TcAC1-3xHA epimastigote protein extracts, using anti-c-Myc antibodies (**E**) to detect TcCARP3 (10% SDS-PAGE) and anti-HA antibodies (**F**) to detect TcAC1 (6% SDS-PAGE). TcCARP3-3xc-Myc expected size: 63.9 kDa. TcAC1-3xHA expected size: 144.2 kDa. Ponceau red staining is shown as loading control.

To further investigate the role of TcAC1, we immunoprecipitated TcAC1-3xHA from total lysates of TcAC1-OE epimastigotes using anti-HA magnetic beads. Then, eluted fractions were analyzed by mass spectrometry. Among the putative TcAC1 interacting partners identified in this analysis, a hypothetical protein of 515 aa (TriTrypDB gene ID: TcYC6_0045920) showed the highest total spectrum count (*P* value = 0.0392, Log_2_ fold change = 5.3750). This protein is encoded by the gene ortholog of *T. brucei* cAMP Response Protein 3 (TbCARP3, gene ID: Tb1125.7.5340), a multi-adenylate cyclase regulator recently characterized in African trypanosomes (11, 36). A list of potential TcAC1 interacting proteins found in this preliminary analysis is shown in Table S2. To study the localization of TcCARP3, we endogenously tagged the protein by CRISPR/Cas9 to generate a TcCARP3-3xc-Myc cell line. Immunofluorescence analysis of these parasites showed a dual localization pattern that resembles that of TcAC1 and TcAC2: contractile vacuole complex and distal flagellar domain (Fig. 8A). Gene tagging of TcCARP3-3xc-Myc was confirmed by PCR and protein expression by western blot analysis (Fig. 8B and C). To confirm the co-localization of TcAC1 and TcCARP3, we transfected TcCARP3-3xc-Myc endogenously tagged epimastigotes with the pTREX-b-TcAC1-3xHA construct, generated for this purpose. This new mutant cell line (named TcCARP3-3xc-Myc/TcAC1-3xHA) was analyzed by immunofluorescence microscopy and western blot (Fig. 8D through F). Our results confirmed the co-localization of TcAC1 (red) and TcCARP3 (green) to the CVC and the flagellar tip of *T. cruzi* epimastigotes, as shown in the merge image (yellow) (Fig. 8D). Co-expression of TcAC1-3xHA (expected size: 144.2 kDa) and TcCARP3-3xc-Myc (expected size: 63.9 kDa) was confirmed by western blot analyses (Fig. 8E and F). In summary, our results indicate that TcAC1 and TcCARP3 share a specific dual localization pattern in the contractile vacuole complex and the flagellar tip of *T. cruzi* epimastigotes, revealing for the first time the presence of two putative cAMP signaling domains in this parasite.

## DISCUSSION

### cAMP microdomains in *T. cruzi*

Extensive evidence has shown that cAMP signaling mediates metacyclogenesis and response to osmotic stress in *T. cruzi* (16 - 22 - 26 - 26 - 47), but the mechanisms underlying these signals have not been elucidated. In this study, we identified different components of cAMP signaling pathways in two putative cAMP microdomains of the parasite: the contractile vacuole complex and the flagellar distal domain. The dual localization pattern of TcAC1, TcAC2, and TcCARP3 has never been observed in other *T. cruzi* proteins. Furthermore, we found two additional adenylate cyclases, TcAC4 and TcAC5, that localized to the CVC. Most importantly, we showed that TcAC1 and TcAC2 are enzymatically active adenylate cyclases, and TcAC1 overexpression led to phenotypes that are intrinsically related to its specific localization: (i) increased cell adhesion, a process that occurs through the tip of the flagellum during metacyclogenesis (5), and (ii) increased tolerance to osmotic stress, a process mediated by the contractile vacuole of the parasite (23 - 49 - 50 - 50). These results are physiologically relevant because sensing the harsh environment in the triatomine hindgut and triggering cell adhesion is prerequisites to complete metacyclogenesis in *T. cruzi* (2, 5, 22). Under these conditions, a response to hyperosmotic stress should be triggered in the parasite to survive. Thereafter, the metacyclic trypomastigote encounters a totally different environment when it invades a mammalian host cell and becomes intracellular, including a rapid decrease in osmolarity (from ∼1,000 to ∼300 mOsm/kg) (2). Regulating the cell volume under these drastic osmotic changes is crucial for parasite survival, and cAMP signals mediate this process (2, 23, 24, 47).

Other components of the cAMP signaling pathway have been reported in these two subcellular compartments: phosphodiesterases B1 and B2 (PDEB1, PDEB2) have been observed in the plasma membrane, concentrated along the flagellum (6, 51, 52), while PDEC2 localizes to the CVC and is involved in osmoregulation (25, 26). *T. cruzi* adenylate cyclases have been previously reported to be flagellar (22, 30). Antibodies raised against the conserved catalytic domain of TcACs label the flagellum and the flagellar pocket of metacyclic trypomastigotes (22). That flagellar pocket localization could be indeed the CVC, because it is difficult to discriminate between these two physically interacting compartments by IFA (47, 53). Here, we confirm the CVC localization of TcAC1 at the ultrastructural level, with no evidence of flagellar pocket localization in any of the micrographs obtained. In addition, endogenously tagged TcCARP3 exhibited the same dual localization as TcAC1. While this manuscript was in preparation, TcCARP3 was identified by mass spectrometry in a *T. cruzi* fraction of enriched flagellar proteins obtained using a proximity-dependent biotinylation approach (54). These authors also reported the localization of this protein at the tip of the flagellum, as confirmed by endogenous tagging and IFA. However, the CVC localization was not observed in these images probably because it is necessary to induce hypoosmotic stress to better visualize this organelle. The presence of all these signaling players in the CVC and the flagellum suggests the existence of at least two cAMP microdomains in *T. cruzi*. These types of signaling microdomains have been previously proposed in *T. brucei* where different components of the cAMP cascade localize to the flagellar tip and flagellum (TbACPs, TbPDEB1, TbCARP3), polarizing the cAMP signals in this Salivarian trypanosome (10 - 14 - 36 - 45).

Membrane microdomains are assembled by lipid rafts, dynamic associations of cholesterol, and sphingolipids that can include or exclude proteins to variable extents (55). These regions of the membrane provide a platform for the aggregation of various signaling proteins through lipid-protein and protein-protein interactions (56). The presence of lipid rafts in the CVC of *T. cruzi* has been described previously (57). The same study demonstrated that the CVC plays a role in transport of (GPI)-anchored proteins to the plasma membrane in a Rab11-mediated fashion. A possible explanation for the dual localization pattern observed in TcAC1 and TcAC2 is that they are targeted to the CVC by endocytic signals and from there to the flagellar membrane via flagellar pocket. Targeting signals to flagellar subdomains have not been identified in trypanosome ACs (45), but assembly of TcAC1 and TcAC2 in lipid rafts could be necessary to maintain their dual localization. In *T. brucei*, CARP3 localization to the flagellar tip is mediated by FLAM8, an axonemal protein that is transported to the tip of the flagellum through the intraflagellar transport (IFT) machinery (58). The interaction of TcAC1 and TcCARP3 in the CVC and flagellar tip should play an important role on their stability in these membrane microdomains.

### The role of cAMP signaling in *T. cruzi*

The role of cAMP signaling in *T. brucei* has been linked to social motility [SoMo, the formation of radial patterns by populations of early procyclic forms on semi-solid surfaces, recently reviewed in reference (12)]. In agreement with this hypothesis, *null* mutants of TbACP5, TbPDEB1, and TbCARP3 showed a defect in colonizing the proventriculus and salivary glands of the tsetse fly (10 - 36 - 36). Furthermore, the role of cAMP in SoMo as a manifestation of pH taxis was recently described (11). However, SoMo has not been reported in *T. cruzi*, a Stercorarian trypanosome that does not cross physical barriers when colonizing the insect vector, but faces other environmental challenges such as nutrient deprivation, changes in ionic composition, acidification of the extracellular milieu, and osmotic stress, when migrating from the midgut to the hindgut of the triatomine vector (2, 3, 47). Stimulation of trypanosome ACs by pH changes is evidenced by their activation in the acidic conditions that *T. brucei* first invaders face during phagocytosis by liver myeloid cells, which inhibits TNF-α synthesis through the cAMP-mediated activation of the host cell PKA (15). *T. cruzi* also faces an acidic environment upon invasion of the mammalian host cell, when trypomastigotes begin to differentiate inside a parasitophorous vacuole, until they are released into the host cell cytosol. Activation of TcACs by pH could be mediating this differentiation process. In this regard, we observed that TcAC1 overexpressing parasites showed a reduced number of amastigotes per infected cell, which could be the consequence of a cAMP imbalance affecting the delicate equilibrium necessary to drive amastigogenesis in these parasites. Cell-derived trypomastigotes overexpressing TcAC1 were also less invasive than control parasites, showing that changes in the normal cAMP content can impair physiological functions in the mammalian host stages (amastigotes and cell-derived trypomastigotes) while stimulating differentiation in the insect forms (metacyclogenesis). Further assays with metacyclic trypomastigotes should be performed to address the role of cAMP in this developmental stage.

The contractile vacuole complex of *T. cruzi* is an organelle specialized in osmoregulation (24, 47). Research from the Docampo lab has extensively shown the CVC role in regulating cell volume under osmotic stress (2, 23 - 59 - 61 - 59 - 61). This group has also described the role of cAMP in RVD (23, 25, 26). Upon hypoosmotic stress, intracellular levels of cAMP increase, triggering the tubulin-dependent migration and fusion of acidocalcisomes to the contractile vacuole, with translocation of TcAQP1 and transfer of osmolytes (2, 23, 24). The presence of phosphodiesterase PDEC2 in the contractile vacuole, whose overexpression impairs osmoregulation (25), supports a model of cAMP-dependent regulatory volume decrease in *T. cruzi*. Our discovery of several TcACs and TcCARP3 in the contractile vacuole of *T. cruzi* is now adding more components to the cAMP signaling cascade that mediates RVD is this parasite. We hypothesize that TcACs could be activated by sensing osmotic stress, which increases cAMP levels in the cytosolic face of the CVC and triggers fusion of acidocalcisomes to this organelle. Water influx to the CVC through TcAQP1 and water release through the physical interaction of the CVC bladder with the flagellar pocket finally allows the recovery of cell volume and ionic composition in the parasite. In RVD assays, we observed a higher tolerance to hypoosmotic stress in epimastigotes overexpressing TcAC1 and TcAC2. Disruption of their localization restored the RVD phenotype to that of control parasites, highlighting the importance of the CVC localization for osmoregulation. Cell adhesion to the culture flask was also altered during *in vitro* metacylogenesis of TcAC1 overexpressing parasites, and altering the localization of the protein restored the normal phenotype. These results suggest that TcAC1 flagellar tip localization is important for parasite adhesion to a substrate (the flask surface), a process that resembles *T. cruzi* adhesion to the hindgut epithelium of the triatomine vector through the flagellar tip during metacylogenesis (5).

While the contractile vacuole is a key structure in *T. cruzi* osmoregulation, this organelle is absent in *T. brucei*. The expansion of its aquaporin repertoire possible facilitated the loss of the CVC as the main osmoregulatory mechanism in *T. brucei* evolution (47). However, in *T. cruzi,* this organelle is present in all developmental stages (23, 49, 50, 53, 57), although its role in osmoregulation has been mainly studied under hypoosmotic stress in epimastigotes. Our results contribute to this evidence by showing that TcAC1 (a CVC-resident protein) is involved in RVD.

The *T. brucei* AC multigene family encodes more than 70 isoforms (12). The expansion and diversification of this gene family have been linked to the extracellular lifestyle of this parasite and to the function of ACs at the host-parasite interface (22). This gene expansion contrasts with intracellular trypanosomatids, such as *T. cruzi* and *Leishmania*, which exhibit a reduced number of these enzymes (29). In the present study, we confirmed that TcAC is a multigenic family by analyzing genomic sequences obtained with more appropriate sequencing and assembly techniques for repetitive genomes.

By expressing HA-tagged versions of TcACs, we showed that at least four TcACs localize to the CVC (TcAC1, TcAC2, TcAC4, and TcAC5), with two of them (TcAC1 and TcAC2) displaying the same dual localization pattern as TcCARP3. The interaction of CARP3 with several ACs (mainly ACP3 and ACP5) at the flagellar tip has been demonstrated in *T. brucei* by immunoprecipitation, proximity labeling, and IFA (11, 36, 62). In addition, ACP5 and CARP3 *null* mutants exhibited the same SoMo defect and were unresponsive to acid pH (9, 11). In *T. brucei*, CARP3 attaches to the inner leaflet of the plasma membrane through N-myristoylation at its N-terminus (36). In this regard, TcCARP3 also has a putative N-myristoylation signal at the N-terminus and could remain attach to the plasma membrane and to the CVC membrane through the same mechanism (12). The fact that CARPs are trypanosome-specific proteins makes them attractive targets for antiparasitic interventions. Identifying the downstream players in this cascade is crucial to understand this PKA-independent signaling pathway and its relevance for parasite survival. Further characterization of TcCARP3 is necessary to elucidate its possible role in cell adhesion, metacyclogenesis, and response to osmotic stress, as well as in other differentiation processes in the life cycle of *T. cruzi*.

### Mechanisms of TcAC activation

Trypanosome ACs comprise multigene families encoding receptor-type proteins with a variable large extracellular N-terminal domain (22, 28, 29). The AC peculiar structure and the absence of G protein-coupled receptors (GPCRs) in trypanosomes led to the hypothesis that these proteins act as receptors in these parasites, activated by extracellular factors (glycosylation, glucose, hormones, other ligands, fever, and pH) through their N-terminal domain (63 - 66). Allosteric activation through the cytosolic region of the protein (C-terminal domain) has been also proposed (67). A third hypothesis is that any stress that generates a membrane perturbation (acidic, proteolytic, thermal, or osmotic stress) leads to the activation of the parasite’s ACs through dimerization of its catalytic domains (22, 65, 68). The mechanism by which ACs sense pH is still unknown, but it has been proposed that alteration in amino acid charges lead to conformational changes that could alter the proportion of enzymatically active AC dimers (12).

Our results indicate that cell adhesion and osmoregulatory capacity are affected in TcAC1 overexpressing parasites. In fact, these processes are triggered when intracellular cAMP levels increase in *T. cruzi* (22, 23), possibly through TcAC activation by an increase in the dimers to monomers ratio in the flagellar tip and CVC membrane. The organization of membrane microdomains is determined by protein and lipidic composition of lipid rafts (55). In mammalian cells, the presence of cAMP signaling components in lipid rafts has been demonstrated using FRET-based biosensors (56). External factors affecting the lipidic composition of membrane rafts (pH, temperature, proteolysis, and hydrophobic interactions) could alter membrane fluidity and the proportion of AC dimers in the microdomain. Other components of this putative cAMP microdomains in *T. cruzi* such as TcCARP3 and PDEs could be sharing spatial distribution with TcAC1 and other TcACs through lipid rafts. In this regard, myristoylation of TbCARP3 is necessary for its localization at the flagellar tip (36), while a FYVE domain is required for PDEC2 localization to the CVC of *T. cruzi* (25).

Hydrophobic interactions between the flagellar tip of the parasite and the triatomine’s rectal cuticle have been described previously (69 - 71). These interactions could alter the composition of this microdomain, activating TcAC1 and TcAC2. In addition, cAMP could be activating or recruiting to the tip of the flagellum a still unidentified membrane protein involved in parasite adhesion to the vector’s hindgut. However, TcAC dimerization/activation could also occur as a consequence of ligand binding through the amino terminal region of the protein or through allosteric regulation of the catalytic domain at the cytosolic face of the membrane (metabolic regulation).

Finally, in *Dyctyostelium discoideum,* adenylate cyclase G (ACG) functions as an intramolecular osmosensor, triggering cAMP synthesis and sporulation in the fruiting bodies under hyperosmotic conditions (72, 73). This enzyme has a similar structure to that of trypanosomal ACs, presenting an N-terminal extracellular region, followed by a single transmembrane domain, and a catalytic domain facing the cytosol of the cell, that is activated by dimerization. However, high osmolality does not activate ACG by inducing its dimerization. Therefore, osmoregulation of DdACG involves a different mode of regulation involving an intramolecular osmosensor domain (72). A similar mechanism could be mediating TcACs activation at the CVC of *T. cruzi,* sensing the osmotic state of the organelle, different to the mechanosensitive channel TcMscS that senses the filling state of the CVC (49, 74). Nevertheless, all these hypotheses should be tested, and further research is necessary to elucidate the specific mechanisms driving the activation of TcACs in each putative cAMP microdomain, and under different environmental conditions.

### Concluding remarks

Our results indicate that cAMP mediates cell adhesion and response to osmotic stress at the flagellar tip and the contractile vacuole complex of *T. cruzi*, respectively. The mechanisms of TcAC1 activation remain unknown but most likely involves TcAC1 dimerization with the consequent synthesis of cAMP, which triggers metacyclogenesis in the hindgut of the triatomine vector. Modulation of TcACs by TcCARP3 could be required under different conditions. *In vivo* studies in the insect vector and in a murine model are needed to further elucidate the role of cAMP in the developmental transitions of the Chagas disease parasite.

## MATERIALS AND METHODS

### Chemicals and reagents

Fetal bovine serum was purchased from R&D Systems (Minneapolis, MN, USA). G418 was from KSE Scientific (Durham, NC, USA). Puromycin, blasticidin S HCl, Subcloning Efficiency DH5α competent cells, BCA Protein Assay Kit, SuperSignal West Pico Chemiluminescent Substrate, Horseradish peroxidase-conjugated anti-mouse and anti-rabbit IgG antibodies, Alexa Fluor 488-conjugated goat anti-mouse, Alexa Fluor 546-conjugated goat anti-rabbit, mouse anti-HA monoclonal antibody, and Pierce Anti-HA Magnetic Beads were from Thermo Fisher Scientific (Waltham, MA, USA). Restriction enzymes, NEBuilder HiFi DNA Assembly Cloning Kit, and Q5 High-Fidelity DNA Polymerase were from New England BioLabs (Ipsich, MA, USA). ZymoPURE Plasmid Miniprep, ZymoPURE II Plasmid Midiprep and DNA Clean & Concentrator-5 were from Zymo Research (Irvine, CA, USA). cAMP-Glo Assay Kit, T4 DNA Ligase, and GoTaq G2 Flexi DNA Polymerase were from Promega (Madison, WI, USA). cOmplete Mini EDTA-free Protease Inhibitor Cocktail was from Roche (Basel, Switzerland). Four millimeters of electroporation cuvettes, Precision Plus Protein Dual Color Standards, and nitrocellulose membranes were from Bio-Rad (Hercules, CA, USA). Mouse anti-c-Myc monoclonal antibody (9E10) was from Santa Cruz Biotechnology (Dallas, TX, USA). Fluoromount-G mounting medium was from Southern Biotech (Birmingham, AL, USA). The pMOTag23M vector (75) was a gift from Dr. Thomas Seebeck (University of Bern, Bern, Switzerland). The pRS315-GPDp-CYCt vector (45) and *S. cerevisiae cyr1-2* mutant (76) were from Dr. Kent Hill (University of California, Los Angeles, CA, USA). Monoclonal antibody against FCaBP was from Dr. David Engman (Northwestern University, Evanston, IL, USA). Polyclonal antibody against TbBiP (44) was from Dr. Jay Bangs (State University of New York, Buffalo, NY, USA). Anti-TcTS antibody (57) was from Dr. Oscar Campetella (Universidad de San Martin, Buenos Aires, Argentina). DNA oligonucleotides were purchased from Integrated DNA Technologies (Coralville, IA, USA). Phenylmethylsulfonyl fluoride (PMSF), N-*p*-tosyl-L-phenylalanine chloromethyl ketone (TPCK), trans-epoxysuccinyl-l-leucylamido-(4-guanidino)butane (E64), protease inhibitor cocktail for use with mammalian cell and tissue extracts (Cat. No. P8340), anti-α-tubulin monoclonal antibody, benzonase nuclease, and all other reagents of analytical grade were from Sigma-Aldrich (St. Louis, MO, USA).

### *In silico* analyses

We searched at TriTrypDB for annotated adenylate cyclases sequences in *T. cruzi* YC6 strain. The retrieved annotated AC sequences were used as queries to search for AC sequences annotated as hypothetical proteins, gene fragments, and pseudogenes using BLASTn tool in tritrypdb.org. This search was also performed for *T. cruzi* Dm28c and TCC strains. These three genome sequences were generated using PacBio and Illumina sequencing technologies (39, 77). Predicted AC amino acid sequences were aligned using ClustalW method in MEGA7 software (78). Evolutionary analyses were conducted in MEGA7 using the Neighbor-Joining method (79) and the bootstrap method with 1,000 replicates (80). The evolutionary distances were computed using the JTT matrix-based method Jones (81). The rate variation among sites was modeled with a gamma distribution (shape parameter = 4). InterProScan run on www.ebi.ac.uk site was used to predict AC domain. Amino acid identity matrices were generated using Clustal Omega tool (www.ebi.ac.uk).

### Cell cultures

*T. cruzi* epimastigotes (Y strain) were grown at 28°C in liver infusion tryptose (LIT) medium (82) supplemented with 10% heat-inactivated fetal bovine serum (FBS), penicillin (100 I.U./mL), and streptomycin (100 µg/mL). Cell density was determined using a Guava Muse Cell Analyzer (Luminex Corporation, Austin, TX, USA). Control parasites transfected with pTREX-n-3xHA empty vector and overexpressing cell lines TcAC1-OE, TcAC2-OE, TcAC3-OE, TcAC4-OE, TcAC5-OE, TcAC1-S, TcAC1-L, TcAC2-S, and TcAC2-L were grown in the presence of 250 µg/mL G418. TcCARP3-3xc-Myc cell line was maintained with 250 µg/mL G418 and 5 µg/mL puromycin. TcCARP3-3xc-Myc/TcAC1-3xHA cell line was grown with 250 µg/mL G418, 5 µg/mL puromycin, and 10 µg/mL blasticidin. Tissue culture-derived trypomastigotes were collected from the culture medium of infected Vero cells, using a modification of the method of Schmatz and Murray (1982) as previously described (83). Vero cells were grown in RPMI supplemented with 10% FBS and maintained at 37°C with 5% CO_2_.

### Overexpression of wild type and mutant TcACs

We amplified by PCR the open reading frames of receptor-type adenylate cyclases TcAC1-TcAC5 (TriTrypDB gene IDs: TcYC6_0015740, TcYC6_0106790, TcYC6_0073070, TcYC6_0107130, and TcYC6_0051460), and truncated versions of TcAC1 and TcAC2 genes in which the non-conserved 3′ end of the gene (L mutants, nt 3614–3846 and nt 3535–3837 for AC1 and AC2, respectively), or the entire region downstream the nucleotide cyclase domain (S mutants, nt 3334–3846 and nt 3268–3837 for AC1 and AC2, respectively) were deleted, using *T. cruzi* Y strain gDNA as template (primers 1–14; Table S2). Then, we cloned these PCR products into pTREX-n-3xHA vector (84) by restriction sites HindIII/XhoI (AC1) and XbaI/XhoI (AC2-5). TcAC3 was also cloned into pTREX-p-3xc-Myc by XbaI/HindIII restriction sites, using pTREX-n-TcAC3-3xHA construct as DNA template for PCR-amplification of the insert (primers 3 and 8; Table S3). pTREX-b-TcAC1-3xHA construct was generated by PCR amplification of AC1-3xHA (primers 15 and 16; Table S3) using pTREX-n-TcAC1-3xHA plasmid as template and then cloning into pTREX-b by HindIII restriction site using NEBuilder HiFi DNA Assembly Cloning Kit (New England Biolabs). Gene cloning was confirmed by sequencing, and constructs were used to transfect *T. cruzi* epimastigotes. Overexpression of wild-type TcACs and mutant proteins TcAC1-S, TcAC1-L, TcAC2-S, and TcAC2-L was confirmed by western blot analysis using anti-HA antibodies. Anti-c-Myc antibody was used to detect TcAC3-3xc-Myc by western blot.

### Endogenous tagging of TcCARP3

We performed a CRISPR/Cas9-mediated endogenous C-terminal tagging of TcCARP3. Briefly, *Trypanosoma cruzi* Y strain epimastigotes constitutively expressing T7 RNA polymerase and SpCas9 were transfected with a T7 sgRNA template obtained by PCR (primers 17 and 18; Table S3) and a donor DNA cassette, amplified from pMOTag23M vector (75) (primers 19 and 20; Table S3). The donor DNA provided to induce homology-directed repair contains a 3xc-Myc tag, a puromycin resistance marker, and 65 and 60-nt homologous regions at the 5′ and 3′ ends of the cassette, respectively. Selection of the protospacer was performed using EuPaGDT (eukaryotic pathogen CRISPR guide RNA/DNA design tool; http://grna.ctegd.uga.edu). We chose a specific sgRNA sequence targeting the 3′ end of TcCARP3 gene (TriTrypDB ID: TcYC6_0045920). Selection of transfectants was done with 5 µg/mL puromycin. Endogenous gene tagging was verified by PCR from gDNA using a specific set of primers (primers 21 and 22; Table S3) and by western blot.

### Transfection of *T. cruzi* epimastigotes

*Trypanosoma cruzi* Y strain epimastigotes were transfected as previously described (84). Briefly, 4 × 10^7^ cells in early exponential phase were washed with PBS pH 7.4 at room temperature (RT) and resuspended in ice-cold CytoMix (120 mM KCl, 0.15 mM CaCl_2_, 10 mM K_2_HPO_4_, 25 mM HEPES, 2 mM EDTA, 5 mM MgCl_2_, pH 7.6) at a final density of 1 × 10^8^ cells/mL. Thereafter, 400 µL of cell suspension was transferred to an iced-cold 4 mm electroporation cuvette containing 25 µg of each DNA fragment (purified plasmid or PCR product) in a maximum DNA volume of 40 μL. Three electric pulses (1,500 V, 25 µF) were applied to the cells in cuvettes, using a Gene Pulser Xcell Electroporation System (Bio-Rad). Transfected epimastigotes were cultured in LIT medium supplemented with 20% heat-inactivated FBS and the corresponding antibiotics for selection of resistant parasites, until stable cell lines were obtained (2–3 weeks). Antibiotic concentrations were 250 µg/mL G418, 10 µg/mL blasticidin, and 5 µg/mL puromycin. Clonal populations of transfectant parasites were obtained by serial dilutions in 96-well plates.

### Western blot analyses

Western blots were performed as previously described (85). Briefly, parasites in exponential phase of growth were washed in PBS and resuspended in radio-immunoprecipitation assay (RIPA) buffer (150 mM NaCl, 20 mM Tris-HCl, pH 7.5, 1 mM EDTA, 1% SDS, 0.1% Triton X-100) plus a mammalian cell protease inhibitor cocktail (diluted 1:250), 1 mM phenylmethylsulfonyl fluoride, 2.5 mM tosyl phenylalanyl chloromethyl ketone, 100 M *N*-(*trans*-epoxysuccinyl)-L-leucine 4-guanidinobutylamide (E64), and benzonase nuclease (25 U/mL culture). Cells were then incubated for 30 minutes on ice, and protein concentration was determined by BCA protein assay. Thirty micrograms of protein from each cell lysate was mixed with 4× Laemmli sample buffer (Bio-Rad) supplemented with 10% β-mercaptoethanol, before application to 8% SDS–polyacrylamide gels. Electrophoresed proteins were transferred onto nitrocellulose membranes with a Trans-Blot Turbo Transfer System (Bio-Rad). Membranes were blocked with 5% nonfat dry milk in PBS-T (PBS containing 0.1% Tween 20) overnight at 4°C. Next, blots were incubated for 1 hour at RT, with the primary antibody: monoclonal anti-HA (1:2,000), monoclonal anti-c-Myc (1:1,000), or monoclonal anti-α-tubulin (1:40,000). After three washes with PBS-T, blots were incubated with the secondary HRP-conjugated antibody (goat anti-mouse IgG or goat anti-rabbit IgG, diluted 1:10,000). Membranes were washed three times with PBS-T and incubated with Pierce ECL Western Blotting Substrate (Thermo Fisher Scientific). Finally, images were acquired and processed with a ChemiDoc Imaging System (Bio-Rad).

### Immunofluorescence analyses

*T. cruzi* parasites (epimastigotes, trypomastigotes, or amastigotes) were washed with PBS and fixed with 4% paraformaldehyde (PFA) in PBS pH 7.4 for 1 hour at RT. IFAs involving TcAC1 and TcCARP3 mutants were performed under hypoosmotic conditions by adding an equal volume of deionized water to the parasites in PBS and fixing them after exactly 2 minutes. Thereafter, cells were allowed to adhere to poly-L-lysine-coated coverslips and then permeabilized for 5 minutes with 0.1% Triton X-100. Then, cells were blocked with trypanosome blocking solution (3% bovine serum albumin [BSA], 1% fish gelatin, 5% normal goat serum, and 50 mM NH_4_Cl in PBS pH 7.4) overnight at 4°C. Cells were then incubated with primary antibody(es): rabbit anti-HA (1:200) and/or mouse anti-c-Myc (1:100), diluted in 1% BSA in PBS (pH 8.0) for 1 hour at RT. Cells were washed three times with 1% BSA in PBS and then incubated for 1 hour at RT with secondary antibody(es): Alexa Fluor 488-conjugated goat-anti mouse (1:1,000) and/or Alexa Fluor 546-conjugated goat anti-rabbit (1:1,000). This incubation was performed keeping the cells protected from light. Then, cells were washed three times with 1% BSA in PBS and mounted on slides using Fluoromount-G mounting medium containing 5 µg/mL 4′,6-diamidino-2-phenylindole (DAPI) to stain DNA. Control IFAs were done as described above but in the absence of a primary antibody. Differential interference contrast (DIC) and fluorescence optical images were captured using an Olympus IX-71 inverted fluorescence microscope with a Photometrix CoolSnap CCD camera driven by DeltaVision Software (Applied Precision, Issaquah, WA, USA). Images were deconvolved for 15 cycles using Softwarx deconvolution software. Images to evaluate TcCARP3 localization and TcCARP3/TcAC1 co-localization were acquired at Cincinnati Children’s Hospital Confocal Imaging Core using a Nikon A1R inverted microscope with NIS-Elements software for image acquisition and processing.

### Electron microscopy

A clonal population of *T. cruzi* parasites expressing TcAC1-3xHA was used for localization of the protein at the ultrastructural level. Epimastigotes, trypomastigotes, and amastigotes were washed twice in 0.1 M sodium cacodylate buffer, pH 7.4, and fixed for 1 hour on ice with 0.1% glutaraldehyde, 4% paraformaldehyde, and 0.1 M sodium cacodylate buffer, pH 7.4. Fixed samples were processed for cryo-immunoelectron microscopy at the Molecular Microbiology Imaging Facility, Washington University School of Medicine (St. Louis, MO, USA). TcAC1 was detected with a polyclonal anti-HA primary antibody and a gold-conjugated anti-rabbit IgG secondary antibody.

### Gene complementation in yeast

For gene complementation in yeast, open reading frames encoding TcAC1–TcAC5 were subcloned from pTREX-n-3xHA vector into the *Saccharomyces cerevisiae* pRS315-GPDp-CYCt expression vector (45), which contains a glyceraldehyde 3-phosphate dehydrogenase (GPD) promoter to allow constitutive high expression, and a cytochrome c isoform 1 (CYC1) terminator. Full-length *T. cruzi* adenylate cyclase coding sequences previously cloned in pTREX-n-3xHA were amplified using forward primers 23–27 (Table S3) and a common reverse primer (primer 28, Table S3) to include the 3xHA epitope tag sequence at the 3′ end of each gene. These open reading frames were subcloned into pRS315-GPDp-CYCt vector using the NEBuilder HiFi DNA Assembly Cloning Kit (New England Biolabs). The resulting plasmids and the empty vector were transformed into the *S. cerevisiae cyr1-2* mutant (76) using standard methods (86). Transformants were selected and maintained on SD-Leu selective medium as described previously (86). Clonal strains were resuspended in PBS at an OD_600_ of 1.0, and four different inocula (10^4^, 10^3^, 10^2^, and 10^1^ cells) obtained by serial dilutions were spotted onto YPD agar plates, and then incubated at permissive (22°C) or restrictive (35°C) temperatures. *S. cerevisiae cyr1-2* mutant, the pRS315-GPDp-CYCt vector, and a control plasmid containing the *S. cerevisiae* wild-type adenylate cyclase (CYR1) coding sequence were kindly provided by Dr. Kent Hill (UCLA).

### Determination of intracellular cAMP

Intracellular levels of cAMP in *T. cruzi* epimastigotes were determined using the luminescent assay cAMP-Glo (Promega) following manufacturer’s protocol. Briefly, *T. cruzi* epimastigotes in exponential phase of growth were washed twice with PBS and resuspended in induction buffer (500 μM 3-isobutyl-1-methylxanthine and 100 μM Ro 20-1724 in PBS, pH 7.4) to a final density of 1 × 10^9^ cells/mL. Then, 10 μL of cell suspension was transferred into a white 96-well plates in triplicates (1 × 10^7^ cells/well). Cells in wells were lysed adding 10 μL of cAMP-Glo lysis buffer and incubating them at RT for 15 minutes. Next, 20 μL of cAMP detection solution was added to each well. Cells in plate were agitated for 1 minute in an orbital shaker and incubated for 20 minutes at RT. Finally, 40μL of Kinase-Glo Reagent was simultaneously added to the wells. After shaking for 1 minute, the plate was incubated for 10 minutes at RT. Luminescence was measured using a BioTek Synergy H1 plate reader (Agilent Technologies, Santa Clara, CA, USA). Results were expressed as mean values of cAMP content relative to control cells from three independent experiments.

### RVD assays

Regulatory volume decrease after hypoosmotic stress was monitored as described previously (49). Briefly, *T. cruzi* epimastigotes in exponential phase of growth were centrifuged at 1,000 × *g* for 7 minutes, washed twice in PBS, and resuspended in isosmotic buffer (64 mM NaCl, 4 mM KCl, 1.8 mM CaCl_2_, 0.53 mM MgCl_2_, 5.5 mM glucose, 150 mM D-mannitol, 5 mM HEPES-Na, pH 7.4, 282 mOsmol/L) at a cell density of 1 × 10^8^ cells/mL. Then, 100 µL of aliquots was placed in a 96-well plate in triplicates and the absorbance at 550 nm was measured every 10 seconds for 3 minutes using a BioTek Synergy H1 plate reader (Agilent Technologies). Immediately, 200 µL of hypoosmotic buffer (64 mM NaCl, 4 mM KCl, 1.8 mM CaCl_2_, 0.53 mM MgCl_2_, 5.5 mM glucose, and 5 mM HEPES-Na, pH 7.4) was added for a final osmolarity of 115 mOsmol/L, and the absorbance at 550 nm was measured after hypoosmotic stress for additional 12 minutes. Readings were normalized against the mean value of the initial 3 minutes in isosmotic buffer. Normalized absorbance readings were then converted into a percent volume change using the following equation: (Vf – Vo/Vo) × 100, where Vf is the absorbance value at the experimental time point and Vo is the absorbance mean value obtained under isosmotic conditions. The osmoregulatory capacity of *T. cruzi* cell lines was quantified using two different parameters: the maximum cell volume change upon induction of hypoosmotic stress (area under the curve between 200 and 300 seconds in the absorbance chart) and the final volume recovery (area under the curve between 700 and 800 seconds).

### *In vitro* metacyclogenesis

Metacyclic trypomastigotes were obtained following the protocol described by Bourguignon et al. (87) with minor modifications. Briefly, *T. cruzi* epimastigotes were cultured for 4 days in LIT medium, washed twice in PBS, resuspended in triatome artificial urine (TAU) medium (190 mM NaCl, 17 mM KCl, 2 mM MgCl_2_, 2 mM CaCl_2_, 0.035% sodium bicarbonate, 8 mM phosphate, pH 6.9), and incubated for 2 hours at RT. Then, parasites were incubated horizontally for 96 hours in TAU 3AAG medium (TAU medium supplemented with 10 mM L-proline, 50 mM sodium L-glutamate, 2 mM sodium L-aspartate, and 10 mM glucose) in T75 flasks. For quantification, assays samples were fixed for 1 hour at RT in 4% PFA in PBS, attached to poly-L-lysine-coated coverslips, and washed three times with PBS. Then, parasites were incubated for 1 hour in 50 mM NH_4_Cl in PBS, washed three more times in PBS, and mounted onto glass slides with Fluoromount-G containing 15 µg/mL DAPI, which stains the DNA present in the nucleus and the kinetoplast of parasites. Twenty fields/slide were analyzed in an Olympus BX60 epifluorescence microscope with a 100× objective in three independent experiments. Metacyclic trypomastigotes were distinguished from epimastigotes by the location of the kinetoplast in the cell body (posterior in metacyclic trypomastigotes; between the nucleus and the flagellum in epimastigotes). An additional step was performed if metacyclic trypomastigotes were obtained to infect Vero cells. To increase the proportion of metacyclic forms, instead of fixing the parasites, the content of the flask was collected and resuspended in RPMI medium containing fresh FBS and then incubated at 37°C for additional 20 hours. The complement in fresh FBS kills epimastigotes, whereas metacyclic trypomastigotes survive. For invasion assays, parasites were harvested after 20-hour incubation in RPMI plus fresh FBS.

### Adhesion assays

During *in vitro* metacyclogenesis, parasites adhere to the flask within the first 6 hours of horizontal incubation in TAU 3AAG medium (22). Subsequently, fully differentiated metacyclic trypomastigotes get detached and are progressively released into the medium throughout the next 96 hours. To quantify the ability of *T. cruzi* epimastigotes to adhere to the flask during the incubation in TAU 3AAG medium, parasite density in the medium was determined at 2, 4, 6, 24, 48, 72, and 96 hours using an automatic cell counter. The number of adhered parasites was obtained by subtracting the total number of non-adhered cells to the initial number of cells added to the flask. The results are expressed as mean values of three independent experiments.

### Host cell invasion and intracellular replication

*T. cruzi* invasion and intracellular replication assays were performed as previously described (88). Briefly, gamma-irradiated (2,000 radiation-absorbed doses) Vero cells (4.5 × 10^5^ cells) were plated onto sterile coverslips in a 12-well plates and incubated overnight at 37°C, 5% CO_2_, in RPMI medium plus 10% fresh FBS. Tissue culture-derived trypomastigotes were incubated at 4°C overnight to allow amastigotes to settle from swimming trypomastigotes. Trypomastigotes from the supernatants of these collections were counted and used to infect Vero cells in the coverslips at a 10:1 multiplicity of infection (MOI). At 4 hours post infection, coverslips were washed extensively with Hank’s balanced salt solution, followed by PBS, pH 7.4, to remove any extracellular parasites. Samples were fixed immediately in 4% paraformaldehyde in PBS, pH 7.4, at 4°C for 30 minutes. Then, coverslips were washed once with PBS and mounted onto glass slides in Fluoromount-G containing 15 µg/mL DAPI, which stains host and parasite DNA. Samples were analyzed on an Olympus BX60 epifluorescence microscope to quantify the number of host cells that contained intracellular parasites and the number of intracellular parasites per cell in randomly selected fields. Three hundred host cells were counted per sample in three independent experiments. To quantify amastigote replication, the following modifications were used: host cells were infected at a MOI of 10, washed extensively with Hank’s balanced salt solution after 4 hours, and incubated for additional 48 hours at 37°C, 5% CO_2_, prior to fixation and DAPI staining. These conditions have been standardized to ensure the invasion of maximum one parasite per host cell. Coverslips were mounted onto glass slides and analyzed by fluorescence microscopy. Amastigotes in infected cells were counted using a 100× objective.

### Immunoprecipitation of TcAC1-3xHA

*Trypanosoma cruzi* epimastigotes (2 × 10^8^ cells) in exponential phase of growth were centrifuged at 1,000 × *g* for 15 minutes and washed twice with buffer A with glucose (BAG; 116 mM NaCl, 5.4 mM KCl, 0.8 mM MgSO_4_, 50 mM HEPES, and 5.5 mM glucose, pH 7.3) at RT. Then, parasites were resuspended in 1 mL ice-cold lysis buffer (0.4% NP-40, 1 mM EDTA, 150 mM KCl, cOmplete Mini EDTA-free Protease Inhibitor Cocktail, 50 mM Tris-HCl, pH 7.5) and mixed for 30 minutes at 4°C on a rocking shaker. Cell lysate was centrifuged at 15,000 × *g* for 20 minutes at 4°C, and the supernatant was incubated with 50 µL of Pierce Anti-HA Magnetic Beads (Thermo Fisher Scientific) previously washed with lysis buffer using a magnetic rack. The soluble fraction of the supernatant was then incubated with magnetic beads for 1 hour at RT under gentle agitation. Magnetic beads were then washed three times with wash buffer (0.1% NP-40, 1 mM EDTA, 150 mM KCl, cOmplete Mini EDTA-free Protease Inhibitor Cocktail, 50 mM Tris-HCl, pH 7.5) using a magnetic rack. TcAC1-3xHA and its interacting proteins were eluted with 100 µL of elution buffer supplied with the beads by applying vortex at a low speed for 10 minutes at RT. Eluates were neutralized with 15 µL neutralization buffer (1M Tris pH 9.5) and analyzed by western blot with anti-HA antibodies. Eluted fractions from TcAC1-3xHA overexpressing parasites and empty vector control cells were sent to the Proteomics Core Facility of the Whitehead Institute (Cambridge, MA, USA) for mass spectrometry analysis.

### Statistical analyses

Values are expressed as mean ± SD. Significant differences between treatments were compared using unpaired Student’s *t*-test, and one-way and two-way ANOVA tests, as indicated in the legend of figures. Differences were considered statistically significant for *P* < 0.05, and *n* refers to the number of independent experiments performed. All statistical analyses were conducted using GraphPad Prism 9 (GraphPad Software, San Diego, CA, USA).

## SUPPLEMENTAL MATERIAL

Figure S1 mbio.01064-23-s0001.pdf Pairwise amino acid sequence identities.

Figure S2 mbio.01064-23-s0002.pdf Phylogenetic tree of TcAC amino acid sequences.

Figure S3 mbio.01064-23-s0003.pdf TcAC clusters in *T. cruzi* YC6 strain chromosomes.

Figure S4 mbio.01064-23-s0004.pdf Phylogenetic tree of TcAC amino acid sequences.

Figure S5 mbio.01064-23-s0005.pdf Dual localization of TcAC1.

Figure S6 mbio.01064-23-s0006.pdf Amino acid sequence alignment.

Figure S7 mbio.01064-23-s0007.pdf Amino acid sequence alignment and iImmunofluorescence analysis.

Table S1 mbio.01064-23-s0008.xlsx Annotation of *T. cruzi* adenylate cyclase (AC) genes and gene fragments.

Table S2 mbio.01064-23-s0009.pdf Potential TcAC1 interacting proteins identified by mass spectrometry.

Table S3 mbio.01064-23-s0010.pdf Oligonucleotides used in this work.

ASM does not own the copyrights to Supplemental Material that may be linked to, or accessed through, an article. The authors have granted ASM a non-exclusive, world-wide license to publish the Supplemental Material files. Please contact the corresponding author directly for reuse.
