# Supplementary material for: Dual localization of receptor-type adenylate cyclases and cAMP response protein 3 unveils the presence of two putative signaling microdomains in Trypanosoma cruzi
Source: mBio. 2023 Jul 21;14(4):e01064-23. doi: 10.1128/mbio.01064-23 (PMC10470820; doi:10.1128/mbio.01064-23)
Supplement: Figure S1 — Pairwise amino acid sequence identities. [file mbio.01064-23-s0001.pdf]

Figure S1

Full-length predicted proteins

	AC1	AC2	AC3	AC4	AC5
AC1	85.1 – 98.7	57.9 – 59.7	59.0 – 60.7	57.6 – 59.0	60.9 – 63.6
AC2		96.6 – 98.2	63.5 – 64.5	67.1 – 68.1	63.0 – 64.1
AC3			98.2 – 99.9	66.2 – 66.5	66.3 – 67.2
AC4				95.1	76.3 – 77.2
AC5					96.4 – 99.9

N-terminal domain

	AC1	AC2	AC3	AC4	AC5
AC1	82.3 – 98.9	54.9 – 55.9	55.0 – 57.8	52.2 – 53.1	56.4 – 57.9
AC2		95.9 – 98.7	63.5 – 65.1	69.0 – 70.4	64.5 – 65.2
AC3			98.0 – 99.9	60.8 – 61.4	61.6 – 63.0
AC4				93.4	74.0 – 75.1
AC5					96.2 – 99.9

Catalytic domain

	AC1	AC2	AC3	AC4	AC5
AC1	92.2 – 100.0	89.6 – 93.5	85.7 – 88.7	89.0 – 90.8	91.8 – 95.2
AC2		98.3– 99.1	87.8 – 89.1	90.4 – 92.6	88.3 – 90.8
AC3			98.7 – 100.0	84.8 – 85.7	84.8 – 86.8
AC4				97.4	86.5 – 89.0
AC5					96.5 – 100.0

C-terminal domain

	AC1	AC2	AC3	AC4	AC5
AC1	80.0 – 100.0	25.4 – 27.3	31.7 – 32.9	38.0 – 39.2	34.8 – 38.1
AC2		95.2 – 97.9	31.3 – 31.8	30.6 – 31.8	26.5 – 29.1
AC3			98.2 – 100.0	70.2 – 70.8	64.9 – 66.7
AC4				99.4 – 100.0	72.6 – 73.2
AC5					97.8 – 100.0

Figure S1. Pairwise amino acid sequence identities among the full-length proteins, and within the N-terminal, catalytic and C-terminal domains of the 17 TcACs found in *T. cruzi* YC6 strain, that were clustered in five groups in this work. AC1: 6 genes, AC2: 2 genes, AC3: 3 genes, AC4: 2 genes, AC5: 3 genes. Multiple sequence alignment was performed with Clustal Omega (www.ebi.ac.uk).
