# Supplementary material for: Dual localization of receptor-type adenylate cyclases and cAMP response protein 3 unveils the presence of two putative signaling microdomains in Trypanosoma cruzi
Source: mBio. 2023 Jul 21;14(4):e01064-23. doi: 10.1128/mbio.01064-23 (PMC10470820; doi:10.1128/mbio.01064-23)
Supplement: Figure S2 — Phylogenetic tree of TcAC amino acid sequences. [file mbio.01064-23-s0002.pdf]

Figure S2

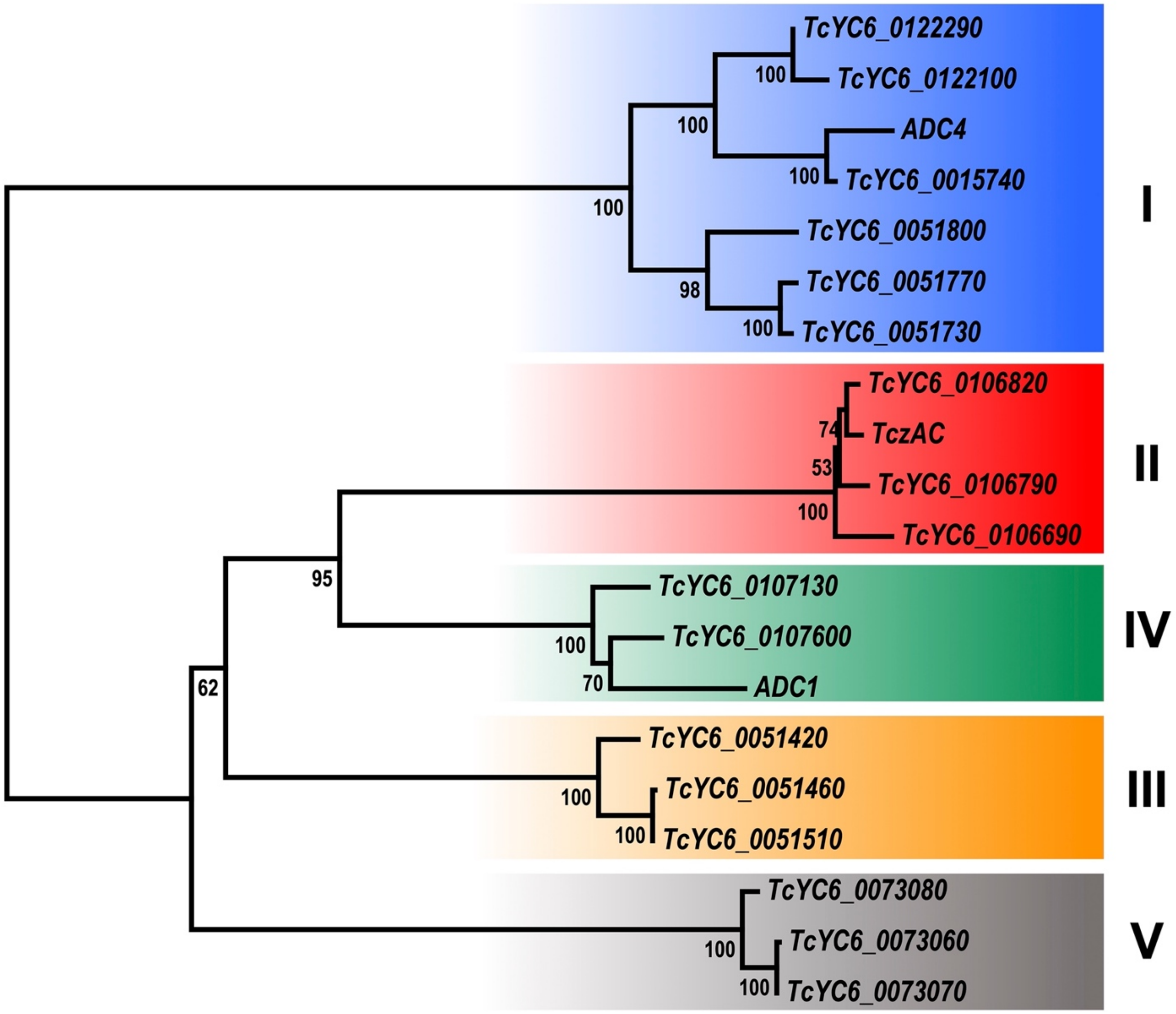

Figure S2. Phylogenetic tree of TcAC amino acid sequences. Phylogenetic tree was constructed using the Neighbor-Joining method with 17 full-length predicted TcAC proteins from *T. cruzi* YC6 strain found in tritrypdb.org. Three TcAC protein sequences from *T. cruzi* CL Brener strain were included: ADC1 (GenBank: AJ012096); ADC4 (AJ011684); TczAC: (AF040382). The tree is drawn to scale, with branch lengths in the same units as those of the evolutionary distances used to infer the phylogenetic tree. Scale is in units of the number of amino acid substitutions per site. Amino acid sequences were aligned using ClustalW method in MEGA7 (Kumar et al., 2016) software. Evolutionary analyses were conducted in MEGA7 (Kumar et al., 2016) using the Neighbor-Joining method (Saitou and Nei, 1987) and the bootstrap method with 1000 replicates (Felsenstein, 1985). The evolutionary distances were computed using the JTT matrix-based method Jones (Jones et al., 1992). The rate variation among sites was modeled with a gamma distribution (shape parameter = 4). Sequences are indicated with the tritrypdb ID. Roman numbers on the right side and shading colors indicate the group of TcAC.
