# Supplementary material for: Dual localization of receptor-type adenylate cyclases and cAMP response protein 3 unveils the presence of two putative signaling microdomains in Trypanosoma cruzi
Source: mBio. 2023 Jul 21;14(4):e01064-23. doi: 10.1128/mbio.01064-23 (PMC10470820; doi:10.1128/mbio.01064-23)
Supplement: Figure S3 — TcAC clusters in T. cruzi YC6 strain chromosomes. [file mbio.01064-23-s0003.pdf]

Figure S3

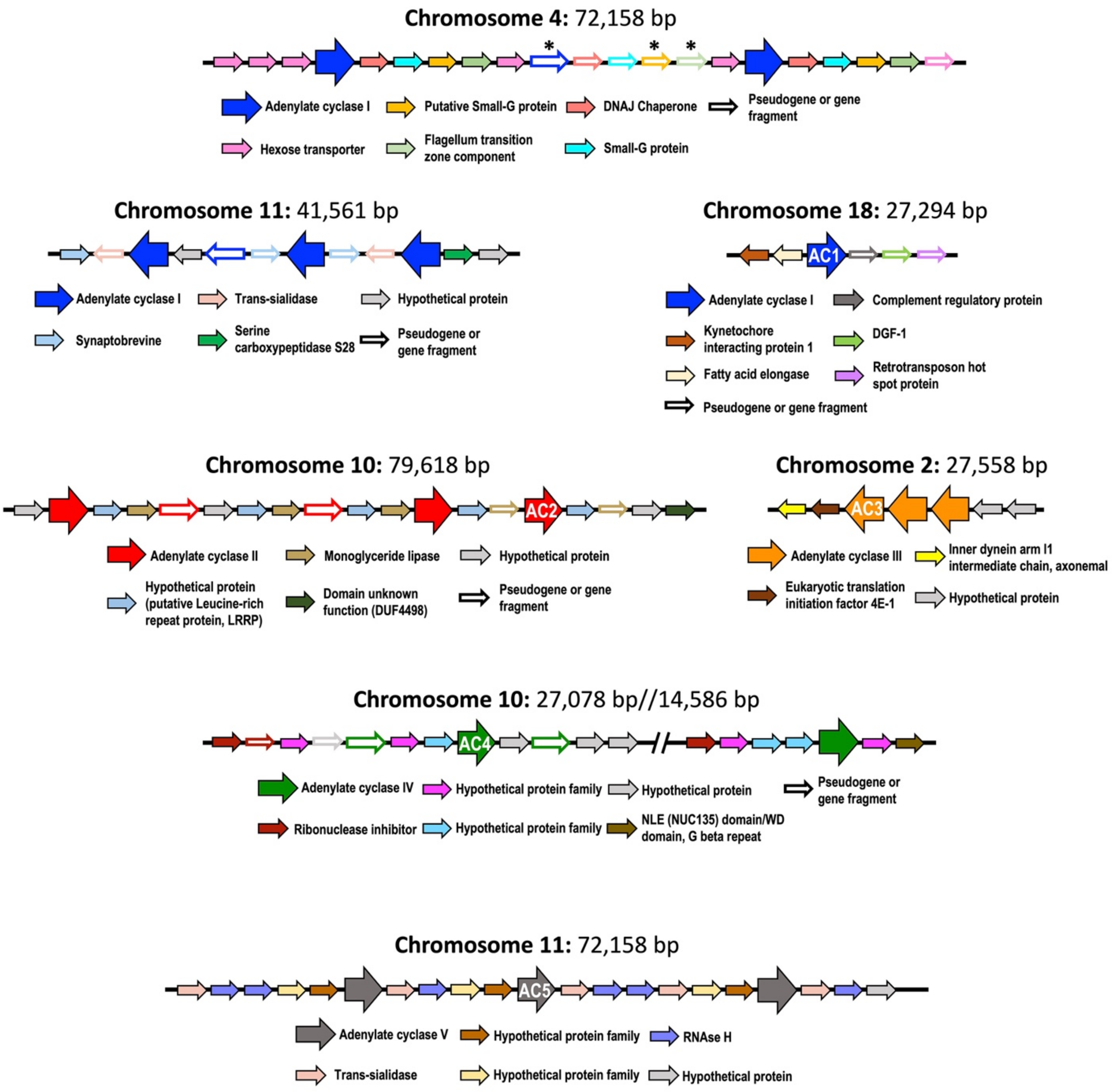

Figure S3. Representation of the TcAC clusters in *T. cruzi* YC6 strain chromosomes. In each chromosome, TcAC copies are shown with big arrows, numbered according to the group to which they belong. Other annotated genes are also shown with small arrows. Empty arrows indicate truncated genes or pseudogenes, whose border color corresponds to the complete genes. Genes are represented from 5' to 3' (genomic), and the arrows point in the predicted transcription direction. Number of bps next to each chromosome indicates the length of the contigs shown in each scheme. The genes (TcAC1-5) that were chosen from each TcAC group in this study are shown in white text within arrows. *Some genomic sequences were manually curated to search for full length and truncated or pseudogenes copies that are represented here and might not match current annotation of TcAC genes in tritrypDB. For more details see Table S1 (data from tritrypdb.org).
