# Supplementary material for: Dual localization of receptor-type adenylate cyclases and cAMP response protein 3 unveils the presence of two putative signaling microdomains in Trypanosoma cruzi
Source: mBio. 2023 Jul 21;14(4):e01064-23. doi: 10.1128/mbio.01064-23 (PMC10470820; doi:10.1128/mbio.01064-23)
Supplement: Figure S4 — Phylogenetic tree of TcAC amino acid sequences. [file mbio.01064-23-s0004.pdf]

Figure S4

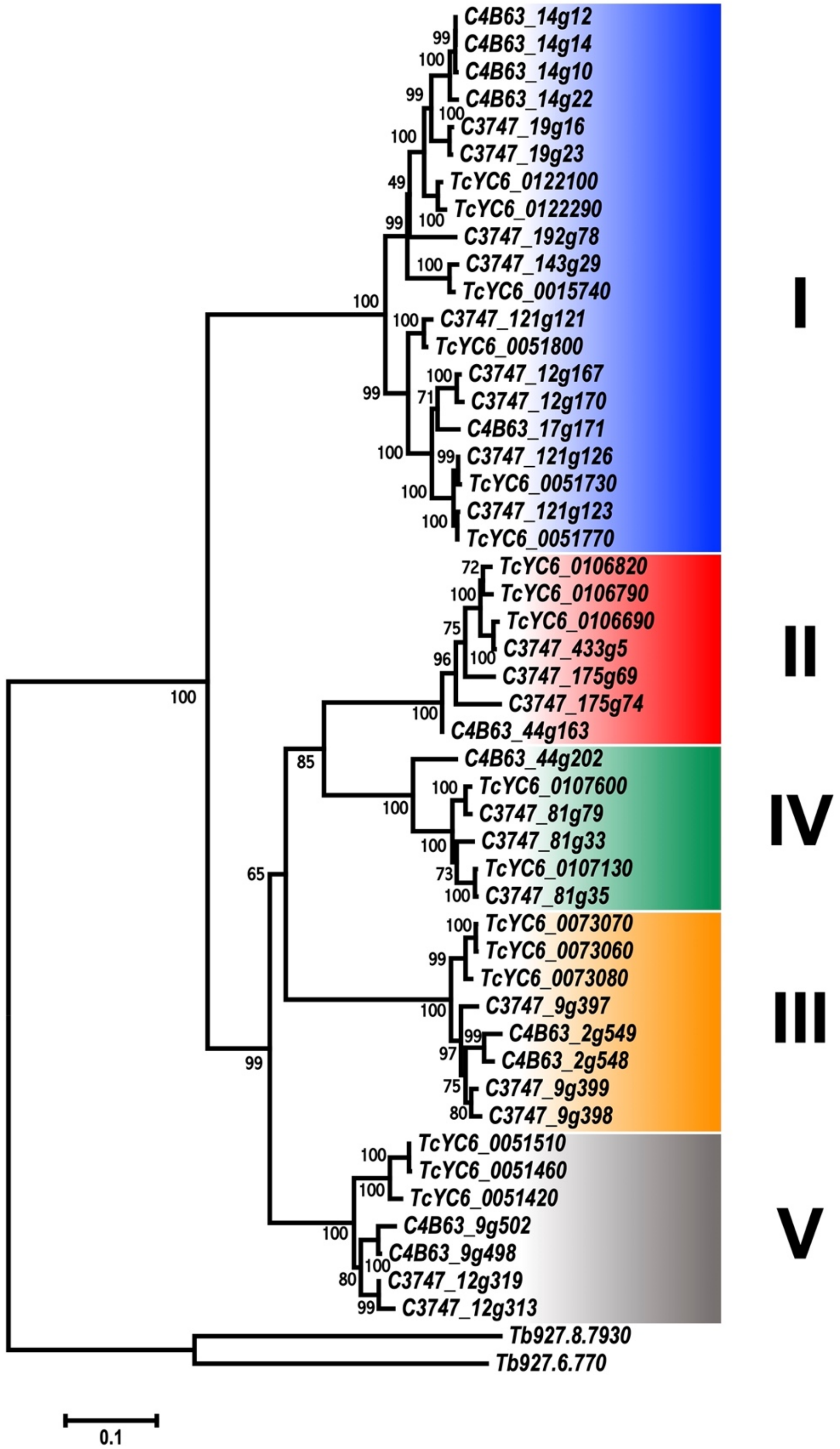
C4B63_14g12
C4B63_14g14
C4B63_14g10
C4B63_14g22
C3747_19g16
C3747_19g23
TcYC6_0122100
TcYC6_0122290
C3747_192g78
C3747_143g29
TcYC6_0015740
C3747_121g121
TcYC6_0051800
C3747_12g167
C3747_12g170
C4B63_17g171
C3747_121g126
TcYC6_0051730
C3747_121g123
TcYC6_0051770
TcYC6_0106820
TcYC6_0106790
TcYC6_0106690
C3747_433g5
C3747_175g69
C3747_175g74
C4B63_44g163
C4B63_44g202
TcYC6_0107600
C3747_81g79
C3747_81g33
TcYC6_0107130
C3747_81g35
TcYC6_0073070
TcYC6_0073060
TcYC6_0073080
C3747_9g397
C4B63_2g549
C4B63_2g548
C3747_9g399
C3747_9g398
TcYC6_0051510
TcYC6_0051460
TcYC6_0051420
C4B63_9g502
C4B63_9g498
C3747_12g319
C3747_12g313
Tb927.8.7930
Tb927.6.770
I
II
IV
III
V
0.1

Figure S4. Phylogenetic tree of TcAC amino acid sequences. Phylogenetic tree was constructed using the Neighbor-Joining method with 17 full-length predicted TcAC proteins from *T. cruzi* YC6 strain, 11 from Dm28c strain, and 20 from TCC strain, found in tritrypdb.org. The tree is drawn to scale, with branch lengths in the same units as those of the evolutionary distances used to infer the phylogenetic tree. Scale is in units of the number of amino acid substitutions per site. Amino acid sequences were aligned using ClustalW method in MEGA7 (Kumar et al., 2016) software. Evolutionary analyses were conducted in MEGA7 (Kumar et al., 2016) using the Neighbor-Joining method (Saitou and Nei, 1987) and the bootstrap method with 1000 replicates (Felsenstein, 1985). The evolutionary distances were computed using the JTT matrix-based method Jones (Jones et al., 1992). The rate variation among sites was modeled with a gamma distribution (shape parameter = 4). Sequences are indicated with the tritrypdb ID (IDs: C4B63 are sequences from *T. cruzi* Dm28c strain, and C3747 are sequences from TCC *T. cruzi* strain*)*. Two *T. brucei* receptor-type adenylate cyclase GRESAG sequences were included as outgroup: tritrypdb ID: Tb927.8.7930 and Tb927.6.770. Roman numbers on the right side and shading colors indicate the group of TcAC.
