# Supplementary material for: Dual localization of receptor-type adenylate cyclases and cAMP response protein 3 unveils the presence of two putative signaling microdomains in Trypanosoma cruzi
Source: mBio. 2023 Jul 21;14(4):e01064-23. doi: 10.1128/mbio.01064-23 (PMC10470820; doi:10.1128/mbio.01064-23)
Supplement: Figure S5 — Dual localization of TcAC1. [file mbio.01064-23-s0005.pdf]

Figure S5

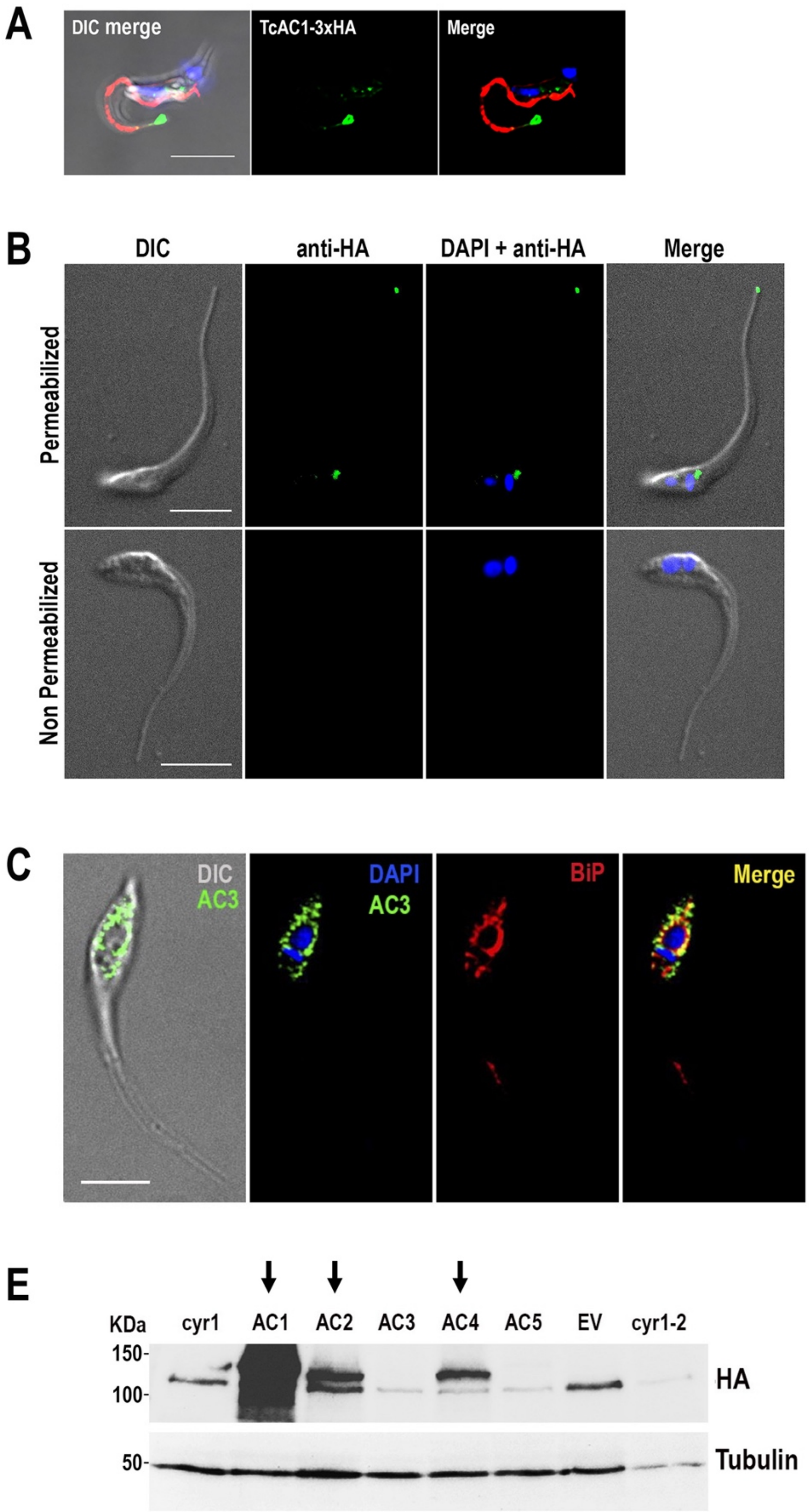
A
DIC merge
TcAC1-3xHA
Merge
B
DIC
anti-HA
DAPI + anti-HA
Merge
Permeabilized
Non Permeabilized
C
DIC
AC3
DAPI
AC3
BiP
Merge
D
kDa
WT
AC3
250
150
100
c-Myc
50
Tub
E
KDa
cyr1
AC1
AC2
AC3
AC4
AC5
EV
cyr1-2
150
100
HA
50
Tubulin

Figure S5. (A) Dual localization of TcAC1 (CVC and flagellar tip) was confirmed by IFA in trypomastigotes expressing TcAC1-3xHA, under hypoosmotic conditions. From left to right the images show differential interference contrast merged with green, red and blue channels (DIC merge), TcAC1-3xHA (green), and merged image including HA labeling (green), DAPI labeling of nucleus and kinetoplast (blue), and flagellar calcium binding protein (FCaBP, red). (B) IFA of permeabilized and non-permeabilized epimastigotes expressing TcAC1-3xHA, using anti-HA antibodies (green). Merged image; DIC, TcAC1 (green) and DAPI (blue). Green labeling was not detected in non-permeabilized parasites. Scale bars: 5 μm. (C) Co-localization of TcAC3 and BiP. Immunofluorescence analysis of TcAC3-3xc-Myc overexpressing epimastigotes using anti c-Myc antibodies (green). BiP, an ER marker, was detected with anti TbBiP antibodies (red). From left to right: DIC and AC3 merged image; DAPI labeling (nucleus and kinetoplast, blue) and AC3 merged image; BiP; AC3, BiP and DAPI merge. Scale bar: 5 μm. (D) Western blot analysis of wild type (WT) and TcAC3-3xc-Myc protein extracts, using anti anti-c-Myc antibodies. α-Tubulin (Tub) was used as loading control. (E) Western blot analysis of total protein extracts from *S. cerevisiae* AC mutant (*cyr1-2*) transformed with a yeast expression vector containing either the yeast wild-type AC (*cyr1*) or the genes encoding *T. cruzi* HA-tagged AC1, AC2, AC3, AC4, and AC5, using anti HA antibodies. An empty vector (EV) yeast and the *cyr1-2* mutant were included in the blot as negative controls. Arrows indicate the successful expression of *T. cruzi* AC1, AC2 and AC4 proteins in yeast. Anti-α-tubulin antibodies were used as a loading control.
