# Supplementary material for: Dual localization of receptor-type adenylate cyclases and cAMP response protein 3 unveils the presence of two putative signaling microdomains in Trypanosoma cruzi
Source: mBio. 2023 Jul 21;14(4):e01064-23. doi: 10.1128/mbio.01064-23 (PMC10470820; doi:10.1128/mbio.01064-23)
Supplement: Figure S6 — Amino acid sequence alignment. [file mbio.01064-23-s0006.pdf]

Figure S6

```
TcYC6_0051800   ...AEREQVEVTALGGVALRGVPAPVEMYQLDAVPGRTFAALRLDRDVPDLDDGSDFESSEDASMY  1131
TcYC6_0051730   ...AEREQVEVTALGGVALRGVPAPVEMYRLDAVPGRTFAALRLDRDVPDLDDGSDFEGGEDVSVY  1094
TcYC6_0051770   ...AEREQVEVTALGGVALRGVPAPVEMYQLDAVPGRTFAALRLDRDVPDLDDGSDFEGGEDVSVY  1131
TcYC6_0015740   ...AEREQVEVTALGGVALRGVPKPVEMYQLDAVPGRTFAALRLDRDVPDLDDGSDFESSEDASMY  1131
TcYC6_0122100   ...AEREQVEVTALGGVALRGVPVPVEMYQLDAVPGRTFAALRLDREMPDLDDGSDFESSEDVSMY  1131
TcYC6_0122290   ...AEREQVEVTALGGVALRGVPVPVEMYQLDAVPGRTFAALRLDREMPDLDDGSDFESSEDVSMY  1131
TcYC6_0106690   ...AEREQVEVTALGAVALRGVPKPVELYQLDAVPGRVVAGLRLDRDDGAVDDYSDCASG-SSVVS  1109
TcYC6_0106790   ...AEREQVEVTALGAVALRGVPKPVEMYQLDAVPGRVVAGLRLDRDDGAVDDYSDCASS-SSGAS  1109
TcYC6_0106820   ...AEREQVEVTALGAVALRGVPKLVEMYQLDAVPGRVVAGLRLDRDDGAVDDYSDCASG-SSVVS  1108
TcYC6_0073080   ...AERKHVEVTALGGVALRGVPKPVEMYQLDAVPGRTFAALRLEREMPVMEEVSDVASG-DGSAS  1109
TcYC6_0073060   ...AERKHVEVTALGGVALRGVPKPVEMYQLDAVPGRTFAALRLEREMPVMEEVSDVASG-DGSVS  1109
TcYC6_0073070   ...AERKHVEVTALGGVALRGVPKPVEMYQLDAVPGRTFAALRLEREMPVMEEVSDVASG-DGSVS  1109
TcYC6_0107130   ...AEREQAEVTALGAVALRGVPKPVEMCQLDAVSGRTFAALRLDREMPDLDDESDLASG-DGSVS  1100
TcYC6_0107600   ...AEREQAEVTALGAVALCGVPKPVEMYQLDAVSGRTFAALRLDREMPDLDDESDLASG-DGSVS  1100
TcYC6_0051420   ...AEREQVEVTALGGVALRGVPKPVEMYRLDAVPGRTFAALRLDREMPDLDDGSDLASG-DGSVS  1104
TcYC6_0051460   ...AEREQLEVTALGGVALRGVPEPVGMYRLDAVPGRTFAALRLDREMPDLDDGSDLASG-DGSVS  1111
TcYC6_0051510   ...AEREQLEVTALGGVALRGVPEPVGMYRLDAVPGRTFAALRLDREMPDLDDGSDLASG-DGSVS  1111
                   ***:: ******.*** ***   * : :**** **..*.***:*:   ::: **  .. .

TcYC6_0051800      SARTGTAQAVVSVLTVTYGTLAPRQRLKALMPLCERWNVRLPRRPSFLQDEEYCRLVIDRLAV  1194
TcYC6_0051730      SARTGTAQAVVSMLTVAYGIFAARQRLKALMPLCERWNVRLPRRPSFVQDEEYCRLVIDRLAV  1157
TcYC6_0051770      SARTGTAQAVVSMLTVAYGIFAARQRLKALMPLCERWNVRLPRRPSFVQDEEYCRLVIDRLAV  1194
TcYC6_0015740      SARTGTAQAVVSVLTVTYGTLAAPQRLKALMPLCERWNVRLPRRPSLVQDEEYCRLVIARLAV  1194
TcYC6_0122100      SARTGTAQAVVSVLTVTYGTLAPRQRLKALMPLCERWNVRLPRRPSFLQDEEYCRLVIDRLAV  1194
TcYC6_0122290      SARTGTAQAVVSVLTVTYGTLAPRQRLKALMPLCERWNVRLPRRPSFLQDEEYCRLVIDRLAV  1194
TcYC6_0106690      RNTCYD--SAVSFISGLMSPYASRQRAGVLESLCRRWRVRVVEK-GVMSYDDYCAALVERLAG  1169
TcYC6_0106790      RNTCYE--SAVSFISGLMSPYASRQRAGVLESLCRRWRVRVVEK-GVMSYDDYCAALVERLAG  1169
TcYC6_0106820      RNTCYD--SAVSFISGLMSPYASRQRAGVLESLCRRWRVRVVEK-GVMSYDDYCAALVERLAG  1168
TcYC6_0073080      TIRGGPSGYVASVLGVLFGTFAAPQRLKALLPLCQRWSVPVPSGAGAGRDKDACRAAMERLAA  1172
TcYC6_0073060      TIRGGPSGYVASVLGVLFGTFAAPQRLKALLPLCQRWSVPVPSGAGAGRDKDACRAAMERLAA  1172
TcYC6_0073070      TIRGGPSGYVASVLGVLFGTFAAPQRLKALLPLCQRWSVPVPSGAGAGRDKDACRAAMERLAA  1172
TcYC6_0107130      TERSGPSGYIASVLAVLFGTFAAPQRLKALFPLCQRWSVRMPRGAGAGCDQDACRVVIGRLAA  1163
TcYC6_0107600      TERSGPSGYIASVLAVLFGTFAAPQRLKALFPLCQRWSVRMPRGAGAGCDQDACRVVIGRLAA  1163
TcYC6_0051420      TDRSGPSGYIASVLAVVFGTFSAPQRLEALLPLCQRWSVPVPSGDGEGREQDACRVAMERLAA  1167
TcYC6_0051460      TDRSGPSGYIASVLAVVFGTFSAPQRLEALLPLCQRWSVPVPSGDGEGREQDACRVAMERLAA  1174
TcYC6_0051510      TDRSGPSGYIASVLAVVFGTFSAPQRLEALLPLCQRWSVPVPSGDGEGREQDACRVAMERLAA  1174
                              .*.:    .  :  **  .*  **.** * :     .    .: *   : ***

TcYC6_0051800      KMSSVMERKGQLGIDQ-NM-SFTLSATAPAAHDTASGTWGRRSGGQSRSRHSNVT--------  1247
TcYC6_0051730      KMSSVMERKGQLGIDQ-NM-SFTLSATAPAAHDTASGTWGRRSGGQSRSRHSNVT--------  1210
TcYC6_0051770      KMSSVMERKGQLGIDQ-NM-SFTLSATAPAAHDTASGTWGRRSGGQSRSRHSNVT--------  1247
TcYC6_0015740      KISNVMERKAQLGMDQ-NM-SFTLSATAPGAHDTASGTWGRRGGEQVHARRFREA--------  1247
TcYC6_0122100      KMSSVMERKGQLGIDQ-NM-SFTLSATAPAAHDTASGTWGRRGGEQVHARRFGEA--------  1247
TcYC6_0122290      KMSSVMERKGQLGIDQ-NM-SFTLSATAPAAHDTASGYVGEEGWGAGPC--------------  1241
TcYC6_0106690      RVSRVIGRRVDGFSGV---SLAEIAATSAVS-----------SQRQSRIRSFQFIGRSGSSSC  1218
TcYC6_0106790      RVSRVIGYRVDGFSGV---SLAEIAATSAVS-----------SQRQSRIRSFQFIGRSGSSSC  1218
TcYC6_0106820      RVGRVIGRRVDGLSGV---SLAEIAAASAVS-----------SQRQSRIRSFQFIGRSGSSSC  1217
TcYC6_0073080      KVCGVIKKRVYGASFEEGLRTLSLSDNASLG-----------STGALRLRNSAFERNGLPVD-  1223
TcYC6_0073060      KVCGVIEKRVYGASFEEGLRTLSLSDNASLG-----------STGALRLRNSAFERNGLPVD-  1223
TcYC6_0073070      KVCGVIEKRVYGASFEEGLRTLSLSDNASLG-----------STGALRLRNSAFERNGLPVD-  1223
TcYC6_0107130      KVCGVIKKRVSGDSIEGGLRTMSLSEGAFIG-----------SDGALRLRRFSVERAGLLGE-  1214
TcYC6_0107600      KVCGVIKKRVSGDSIEGGLRTMSLSEGAFIG-----------SDGALRLRRFSVERAGLLGE-  1214
TcYC6_0051420      KLCGVLKKRASEVSVGEGLRTVHLSKNTLLW-----------SDGALRSRNFSVEGAGLLGE-  1218
TcYC6_0051460      KVCGVLKKRASEVSVGEGLRTMHLSKNTLLW-----------SDGALRSRNFSVEGAGLLGE-  1225
TcYC6_0051510      KVCGVLKKRASEVSVGEGLRTMHLSKNTLLW-----------SDGALRSRNFSVEGAGLLGE-  1225
                   ::  *:  :               ::  :                .

TcYC6_0051800      ---------------VSQSN---SVDL-HNFTADEPRFSTDDSIVTVRAR------RFR--   1281
TcYC6_0051730      ---------------VSQSN---SVDL-HNFTADEPRFSTDDSTVTVRAR------RFR--   1244
TcYC6_0051770      ---------------VSQSN---SVDL-HNFTADEPRFSTDDSTVTVRAR------RFR--   1281
TcYC6_0015740      ---------------APQSN---SVEV-HNFAADEPQFLTDDSVVTVRAR------RFR--   1281
TcYC6_0122100      ---------------ASQSN---SVEV-HAFTADEPRFSTDDSIVTVRAR------RFR--   1281
TcYC6_0122290      -------------------------------------------------------------   1241
TcYC6_0106690      TELSSVVPPVDGSEALPQESLVCPIVMRPTVLRELGWRVHVSSVTSYSDSLDPDAMRPRGT   1279
TcYC6_0106790      TELSSVVPPVDGSEALPQESLVCPIVMRPTVLRELGWRVHVSSVTSYSDSLDPDAMRPRGT   1279
TcYC6_0106820      TELSSVVSPVDGSEALPQESLVCPIVMRPTVLRELGWRVHVSSVTSYSDSLDPDAMRPRGT   1278
TcYC6_0073080      ---------FRRRSGLRQASLSA---------VGEAWPAEEDPVATIHVR------RPV--   1258
TcYC6_0073060      ---------FRRRSGLRQASLSA---------VGEAWPAEEDPVATIHVC------RPV--   1258
TcYC6_0073070      ---------FRRRSGLRQASLSA---------VGEAWPAEEDPVATIHVC------RPV--   1258
TcYC6_0107130      ---------LHRRSGLRQASLSS---------AEEVWWVEEDPVATIQVR------RRV--   1249
TcYC6_0107600      ---------LHRRSGLRQASLST---------AEEVWWVEEDPVATIQVR------RRV--   1249
TcYC6_0051420      ---------SN--------------------------------------------------   1220
TcYC6_0051460      ---------SHWRGGMRQASLST---------AEELWWAEGDSVTTIRVR------RLA--   1260
TcYC6_0051510      ---------SHWRGGMRQASLST---------AEELWWAEGDSVTTIRVR------RLA--   1260
```

Figure S6. Amino acid sequence alignment of the C-terminal region of the 17 TcAC genes found in *T. cruzi* YC6 strain (tritrypdb.org). Color of gene IDs are on the left indicate the group of TcAC: Blue, AC I; red, AC II; yellow, AC III; green, AC IV; grey, AC V. Color bars above the aligned sequences indicate: catalytic domain (burgundy), proximal C-terminal domain (blue); distal C-terminal domain (light blue). (*****) Fully conserved residue; (**:**) residues with strong similar properties; (**.**) residues with weak similar properties.
