# Supplementary material for: Dual localization of receptor-type adenylate cyclases and cAMP response protein 3 unveils the presence of two putative signaling microdomains in Trypanosoma cruzi
Source: mBio. 2023 Jul 21;14(4):e01064-23. doi: 10.1128/mbio.01064-23 (PMC10470820; doi:10.1128/mbio.01064-23)
Supplement: Figure S7 — Amino acid sequence alignment and iImmunofluorescence analysis. [file mbio.01064-23-s0007.pdf]

Figure S7

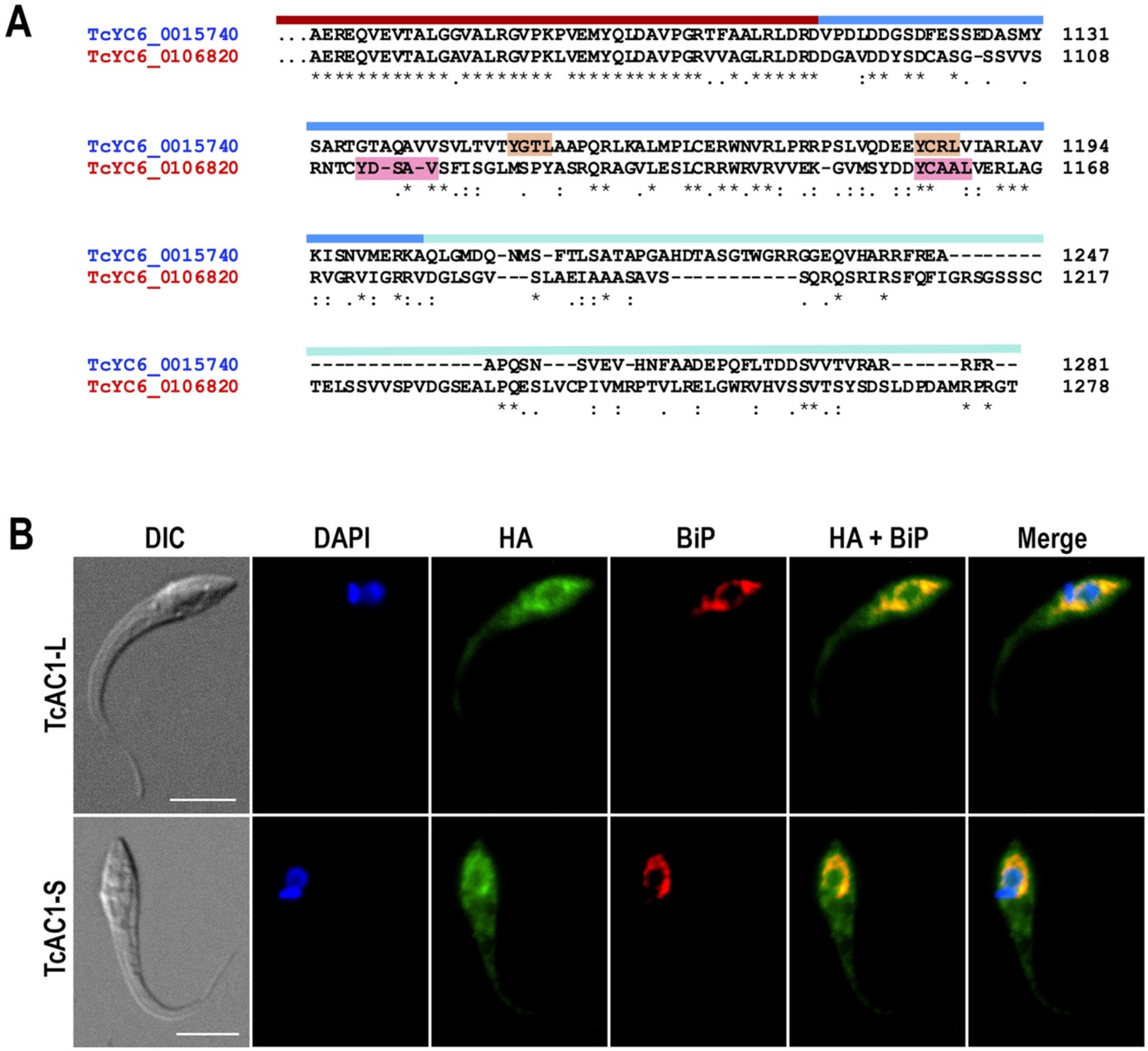

Figure S7. (A) Amino acid sequence alignment of the C-terminal region of TcAC1 and TcAC2 genes found in *T. cruzi* YC6 strain (tritrypdb.org). Gene ID colors on the left indicate the group of TcAC: Blue, ACI; red, ACII. Color bars above the aligned sequences indicate: catalytic domain (burgundy), proximal C-terminal domain (blue); distal C-terminal domain (light blue). Enclosed in squares are putative endocytic motifs: YXXΦ (orange); YXXXΦ (pink). Φ: bulky hydrophobic residue: L/I/M/V/F; (*****) Fully conserved residue; (**:**) residues with strong similar properties; (**.**) residues with weak similar properties. (B) Immunofluorescence analysis of TcAC1-L and TcAC1-S mutants using anti-HA and anti-BiP antibodies. From left to right: DIC, DAPI (blue), HA (green), BiP (red), and merged images. Scale bars: 5 μm.
