# Supplementary material for: Dual localization of receptor-type adenylate cyclases and cAMP response protein 3 unveils the presence of two putative signaling microdomains in Trypanosoma cruzi
Source: mBio. 2023 Jul 21;14(4):e01064-23. doi: 10.1128/mbio.01064-23 (PMC10470820; doi:10.1128/mbio.01064-23)
Supplement: Table S2 — Potential TcAC1 interacting proteins identified by mass spectrometry. [file mbio.01064-23-s0009.pdf]

Table S2. List of potential TcAC1 interacting proteins identified by mass spectrometry.

Gene ID*	Description	MW (kDa)	FC (log_2)**	P value	GO Terms
TcYC6_0015740[#]	Receptor-type adenylate cyclase	140.7	6.9069	0.0321	Cyclic nucleotide biosynthetic process; intracellular signal transduction
TcYC6_0045920	cAMP response protein 3	58.2	5.3750	0.0392	Protein binding
TcYC6_0109520	Hypothetical protein, conserved	74.9	3.6439	0.0277	No data available
TcYC6_0126490	ATP-dependent RNA helicase DBP2A, putative	66.9	0.9386	0.0303	Nucleic acid binding; helicase activity; ATP binding
TcYC6_0106330	Thiolase protein-like protein, putative	48.1	0.7655	0.0424	Acyltransferase activity; acyltransferase activity, transferring groups other than amino-acyl groups
TcYC6_0127880	Glutamate dehydrogenase, putative	48.8	0.6845	0.0443	Cellular amino acid metabolic process; obsolete oxidation-reduction process; oxidoreductase activity; oxidoreductase activity, acting on the CH-NH2 group of donors, NAD or NADP as acceptor
TcYC6_0028110	Tryparedoxin peroxidase, putative	22.4	0.6189	0.0465	Obsolete oxidation-reduction process; peroxidase activity; antioxidant activity; oxidoreductase activity; peroxiredoxin activity
TcYC6_0034780	Replication Factor A 28 kDa subunit, putative	28.3	0.5850	0.0489	DNA replication; DNA repair; DNA recombination; nucleus; nucleic acid binding; DNA binding

* Gene ID in TriTrypDB.
** Log_2 fold change. Significantly abundant proteins showing a P value < 0.05 and a Log_2 fold change ≥ 0.5 (cutoff for fold change = 2-fold) were included in this list.
[#] TcAC1 is the most abundant protein in the eluate (bait).
