# Supplementary material for: Dual localization of receptor-type adenylate cyclases and cAMP response protein 3 unveils the presence of two putative signaling microdomains in Trypanosoma cruzi
Source: mBio. 2023 Jul 21;14(4):e01064-23. doi: 10.1128/mbio.01064-23 (PMC10470820; doi:10.1128/mbio.01064-23)
Supplement: Table S3 — Oligonucleotides used in this work. [file mbio.01064-23-s0010.pdf]

Table S3. Oligonucleotides used in this work.

N°	Primer name	Sequence (5′ → 3′)
1	**FwAC1-HindIII**	CTATAAGCTTATGGTCACGGGATGGGCGGT
2	**FwAC2-XbaI**	TATATCTAGAATGGCGGTGGGATGGGTGGC
3	**FwAC3-XbaI**	TATATCTAGAATGGCGGTGGGGGGATG
4	**FwAC4-XbaI**	TATATCTAGAATGGCGATGAGGTGGGTTGC
5	**FwAC5-XbaI**	TATATCTAGAATGGCGGTGAGGTGGGTGAC
6	**RvAC1-XhoI**	GATTCTCGAGCCTGAAGCGACGAGCAC
7	**RvAC2-XhoI**	ATATCTCGAGTGTGCCTCGCGGCCGCATAGC
8	**RvAC3-XhoI**	ATATCTCGAGCACTGGCCGACAAACGTGA
9	**RvAC4-XhoI**	ATATCTCGAGCACACGCCGGCGAACTTGAA
10	**RvAC5-XhoI**	ATATCTCGAGCGCAAGCCGACGAACGCGA
11	**RvAC1-CTerL-XhoI**	ACTGCTCGAGAGCCTTCCGTTCCATCACG
12	**RvAC1-CTerS-XhoI**	ACTGCTCGAGACGATCAAGACGCAGCGCGGC
13	**RvAC2-CTerL-XhoI**	ACTGCTCGAGTAAACGGCGCCCAATAACACGG
14	**RvAC2-CTerS-XhoI**	ACTGCTCGAGCCTATCAAGCCTCAGCCCAGCC
15	**FwAC1-3xHA-HindIII**	TTGTCTAGAATTCGATATCAATGGTCACGGGATGGGCG

**16**	**RvAC1-3xHA-HindIII**	CGAGGTCGACGGTATCGATATGCCTTGGAGTCGTAAATGGC
**17**	**FwsgRNA_CARP3-CTag**	GAAATTAATACGACTCACTATAGGCTCCCTCTTTATGATGTTTGGTTTTAGAGCTAGAAATAGC
**18**	**RvG00**	AAAAGCACCGACTCGGTGCCACTTTTTCAAGTTGATAACGGACTAGCCTTATTTTAACTTGCTATTTC TAGCTCTAAAAC
**19**	**FwCARP3_CTag ultramer**	GCATCATGCGGTCTGTGATGGAGTCCCGCAAGCGTGGCGGCTCCCTCTTTATGATGTTTGAAGATGGT ACCGGGCCCCCCCTCGAG
**20**	**RvCARP3_CTag ultramer**	CGAGTTCTTTTGGCTCATGTATCATTTGCGTACATCATCATCATCATCATCGTCATCGGGTGGCGGCC GCTCTAGAACTAGTGGAT
**21**	**FwCARP3_CTag check**	AGCCGACGTGCAGTTTGAAC
**22**	**RvCARP3_CTag check**	GAGCCAAAAGAACTCGCCATGA
**23**	**FwAC1_pRS315**	GACGGATTCTAGAACTAGTGATGGTCACGGGATGGGCG
**24**	**FwAC2_pRS315**	GACGGATTCTAGAACTAGTGATGGCGGTGGGATGGGTG
**25**	**FwAC3_pRS315**	GACGGATTCTAGAACTAGTGATGGCGGTGGGGGGATGG
**26**	**FwAC4_pRS315**	GACGGATTCTAGAACTAGTGATGGCGATGAGGTGGGTTGC
**27**	**FwAC5_pRS315**	GACGGATTCTAGAACTAGTGATGGCGGTGAGGTGGGTGAC
**28**	**RvAC1-5_pRS315**	AGGGGCCTGTTTACTCGAGGGTTGCCTTGGAGTCGTAAATGGC
